# Acknowledgement to Reviewers of the *International Journal of Molecular Sciences* in 2014

**DOI:** 10.3390/ijms16011336

**Published:** 2015-01-07

**Authors:** IJMS Editorial Office

**Affiliations:** MDPI AG, Klybeckstrasse 64, CH-4057 Basel, Switzerland

The editors of the *International Journal of Molecular Sciences* would like to express their sincere gratitude to the following reviewers for assessing manuscripts in 2014:

Abass, Khaled
Abbott, David H.
Abdelmohsen, Kotb
Abdraboh, Mohamed
Abe, Naohito
Abe, Toshiaki
Abou Neel, Ensanya A.
Abou-Alfa, Ghassan
Abraini, Jacques H.
Abram, Florence
Abusco, Anna Scotto
Abzalimov, Rinat
Ackland, K.
Acuna-Castroviejo, Darío
Acunzo, M.
Adamcakova-Dodd, Andrea
Adeniji, Adegoke
Adessi, Alessandra
Adhikary, Amitava
Afseth, Nils
Agalliu, Dritan
Agarwal, Anika
Ageorges, Agnès
Agorastos, Agorastos
Agostini, Marco
Agterberg, Martijn J. H.
Aguilar, Claudio
Aguilar-Reina, José
Agulló-Ortuño, M. Teresa
Agyei, Dominic
Ahmed, Khalil
Ahmed, Salahuddin
Ahn, Joong-Hoon
Ahn, Suk-Kyun
Aibner, Thomas
Aizawa, Shin-Ichi
Ajikumar, Parayil Kumaran
Akashi, Makoto
Akbar, Sheikh Mohammad Fazle
Akbarzadeh, A. H.
Akeda, Koji
Akimoto, Jun
Aktas, C.
Akue, Jean Paul
Al Ghouleh, Imad
Al-Ahmadie, Hikmat A.
Alber, Birgit E.
Albero, Cenci
Alcaro, Stefano
Alemán, José
Aleman, Tomas S.
Alexander, David Earl
Alexander, Stephanie
Alexiou, Christoph
Alexis, Frank
Alexopoulos, A.
Alfandari, Dominique
Alhede, Morten
Alisi, Anna
Alison, Malcolm R.
Al-Kateb, Huda
Allen, Randy
Aller, Patricio
Almagro, Lorena
Almeida, Raquel
Alonso-Nanclares, Lidia
Alpini, Gianfranco
Alric, Jean
Altenbach, Susan B.
Altmann, André
Alvarez Ponces, David
Alvarez-Suarez, José M.
Alves, Artur
Amacher, David E.
Amado, Francisco
Amarasena, Najith
Amelia, Matteo
Amin, Kawa
Amir, Rachel
Amital, Howard
Ammirati, Mario
Amoaku, Winfried M.
Anastasi, Emanuela
Anders, Hans-Joachim
Andersen, T. L.
Anderson, Jon P.
Anderson, Julie A.
Anderson, Travis M.
Andia, Isabel
Andjelkovic, Anuska V.
Andolfi, Anna
Andrade, Fernando
Andrade, Paula Andrade
Andrechek, Er
Andreucci, Michele
Andric, Silvana A.
Andújar, Isabel
Angelini, Corrado
Angelini, Sabrina
Angelucci, Adriano
Angiolella, Letizia
Angkawidjaja, Clement
Angulo, Ana
Anifandis, Georgios M
Anna Maria, Giammarioli
Annaert, Wim
Ansquer, Jean Claude
Antonella, Piozzi
Antoni, Delphine
Antonio, Baltazar
Ao, Sio-Iong
Aquino, Rita Patrizia
Aranda, P.
Arcangeli, Annarosa
Arcari, Paolo
Arce, Víctor M.
Arceci, Robert J.
Archer, Trevor
Arduino, Paolo G.
Arens, Ramon
Ariga, Hiroyoshi
Ariga, Katsuhiko
Ariunbaatar, Javkhlan
Ariyama, Kaoru
Arnason, John T.
Arnhold, Juergen
Arnold, Julia T.
Arnold, Shanna
Arnoldi, Anna
Aroca, Ricardo
Arpicco, Silvia
Arteaga, Jesús F.
Artese, Anna
Aruoma, Okezie
Aschberger, Karin
Ashby, Richard D.
Ashida, Hitoshi
Assimakopoulos, Stelios
Astolfi, Stefania
Atrens, Andrej
Auer, Carol
Auron, Philip E.
Austriaco, Nicanor
Avato, Pinarosa
Avila, Matias A.
Avvedimento, Enrico V.
Axelsen, Martin
Aydi, Samir
Aye, I. L. M. H.
Aykin-Burns, Nukhet
Azad, Abul K.
Azmi, Asfar
Baarsma, Hoeke
Baba, Hideo
Baba, Otto
Babu, Uma
Baczek, Tomasz
Bae, Jeoung Won
Bae, Jong-Sup
Baeg, Jin-Ook
Baek, Jeong-Hwa
Baek, Kwang-Hyun
Baele, Guy
Baergen, Rebecca N.
Baffy, György
Bagby, Michael
Bagella, Luigi
Bailly, Sabine
Baino, Francesco
Bak, Min-Ji
Bakare, Oladapo
Baklouti, Faouzi
Bakshi, Mandeep Singh
Balandin, Alexander A.
Bald, Dirk
Ballhorn, Daniel J.
Ballmer-Hofer, Kurt
Baluska, Frantisek
Bandiera, Stelvio M.
Banerjee, Debabrata
Banerjee, Sanjeev
Bano, Daniele
Banskota, Arjun H.
Bao, Bin
Baptista, Pedro V.
Baraldi, Pier Giovanni
Baranov, Pavel V.
Baratta, Mario
Barbe, Mary F.
Barbeyron, Tristan Barbeyron
Barbosa-Pereira, Letricia
Bard, Frederic
Barderas, Maria G.
Bardoni, Barbara
Barnard, K. R.
Barnes, D. W.
Barresi, V.
Barrett, Kim E.
Barriere, Yves
Barros, Pedro
Barroso, Margarida
Barta, Andrea
Bartlett, David L.
Bartlett, John
Bartlett, Michael
Bartoloni, Giovanni
Baruscotti, Mirko
Barz, Bogdan
Basaraba, Randall J.
Basco, Leonardo K.
Basini, Giuseppina
Basnet, Purusotam
Bassareo, Pier Paolo
Bassil, Nahla V.
Bassols, Anna
Bast, Robert
Bastiaan-Net, Shanna
Bastola, Dhundy R.
Basu, Partha
Batail, Patrick
Bates, David
Batista, Frederico M.
Batistuzzo, Camila
Batoko, Henri
Batra, Jyotsna
Battino, Maurizio
Bauer, Johann
Baugé, Catherine
Baulieu, Etienne-Emile
Bayer, Thomas A.
Bayless, Kayla
Baylis, Chris
Bazhin, Alexandr
Beasley, Shea
Beaulieu, Lea M.
Beauregard, Marc
Bechter, Karl
Becker, Therese M.
Bedrosian, Tracy A.
Behrends, Soenke
Beiko, Robert
Beischlag, Timothy V.
Beisswenger, Christoph
Bejai, Sarosh
Bell, Diana
Bellincampi, Daniela
Bellinger, Rick
Bello, Xanel Vecino
Belousov, A. B.
Ben-Dov, Iddo Z.
Benfenati, Emilio
Benfenati, Fabio
Benhabib, Karim
Benjamim, Claudia Farias
Bennett, Barbara K.
Bennett, Gary
Benter, Thorsten
Bentley, T. William
Benton, Richard L.
Beraza, Naiara
Berben, Gilbert
Berber, Mohamed Reda
Bergamaschi, Enrico
Berger, Peter
Berger, Ralf G.
Bergeron, G.
Bermudez, H.
Bernardo, Enrico
Bernhardt, Anne
Bernstein, Hans-Gert
Berrier, Allison L.
Berry, Jody D.
Bersuker, Isaac B
Berteau, Olivier
Bertin, Annabelle
Bertolini, Francesco
Besalú, Emili
Bessho, Yoshitaka
Bester, Megan J.
Beveridge, Thomas J. R.
Beverly, Levi J.
Beynon, Christopher
Beyssac, Eric
Bezrukov, Sergey
Bhatia, Sujata
Bhatnagar, Vibha
Bhushan, Alok
Bhutani, Nidhi
Bi, Enguang
Biamonti, Giuseppe
Bian, Zhaoxiang
Bianco, Roberto
Bidère, Nicolas
Bielenica, Anna
Bignami, Margherita
Bility, Moses
Binder, Harald
Bisaglia, Marco
Biscetti, F.
Biswas, Amlan
Biswas, Arijit
Biswas-Fiss, Esther E.
Bita, Craita E.
Bizzarri, Mariano
Blackstone, Craig
Blanca, Miguel
Blanco, Francisco J.
Blanco, Ignazio
Blanco, José L. Jiménez
Blattmann, C.
Bloch, Wilhelm
Block, Jean-Claude
Blossom, Sarah J.
Blot, Stijn
Blum, Amy
Blum, Marc-Michael
Blume, Cornelia
Boccafoschi, Francesca
Bock, Dan G.
Bode, Konrad
Boelsterli, Urs A.
Bogdanove, Adam
Bohm, Markus
Böhmer, Frank-D.
Böhne, Astrid
Boikos, Sosipatros
Bolitho, Megan
Bonaventura, Giovanni Di
Bonay, M.
Bondos, Sarah E.
Boneca, Ivo G.
Bonetti, Bruno
Bonilla, Carolina
Bonnefont-Rousselot, Dominique
Bonner, William
Bonora, Elena
Boon, Wah Chin
Boor, Peter
Boos, A. M.
Borea, Pier Andrea
Borges, Frédéric
Borlee, Brad
Borsani, Elisa
Bortolus, Renata
Boss, Paul K.
Bosso, Luciano
Bot, Ilze
Botchkareva, Natalia V.
Botella, Miguel A.
Bothner, Brian
Bouabdallah, Ibrahim
Boucrot, Emmanuel
Bouma, Gerrit J.
Bousbaa, Hassan
Boutin, Jean
Boykin, Laura M.
Bracken, Cameron P.
Braig, Simone
Brait, Mariana
Brambrink, A.
Brandao, Lucas
Braun, Thomas
Bräuner-Osborne, Hans
Brebu, Mihai
Breitbart, Haim
Breitkopf-Heinlein, Katja
Brendlé, Jocelyne
Brenner, Ingrid
Breton, Timothy
Brewer, George J.
Brewster, Lizzy
Breydo, Leonid
Brigelius-Flohé, Regina
Brighty, David
Brini, Marisa
Brito, Maria Alexandra
Brizzi, Maria Felice
Broberg Palmgren, Karin
Brodsky, Alexander S.
Broeckling, Corey
Broggini, Massimo
Bronisz, Agnieszka
Brooks, Andrew J.
Brooks, Donald E.
Brosens, Jan J.
Brotto, Marco
Brown, Gregory
Brown, Lindsay
Brown, Mark A.
Bruce, Kimberley D.
Bruce, Lesley J.
Bruce, W. R.
Bruckmaier, R. M.
Brufau, Joaquim
Brumm, Phillip J.
Bruneau, Christian
Brunel, Jean
Brunelle, Alain
Brunelleschi, Sandra
Bruner-Tran, Kaylon L.
Brunham, Liam R.
Brüning, Ansgar
Brunner, Michael
Bruno, John
Bruno, Richard S.
Bruserud, Øystein
Bryan, Jeffrey
Bubulya, Paula A.
Buchman, Vladimir L.
Buechler, Christa
Buehler, Paul W.
Bunaciu, Rodica
Buonaccorsi, Vincent P.
Burdon, Kathryn P.
Burgers, Peter C.
Burgess, Karl E. V.
Burgoon, Mark P.
Burgoyne, Joseph Robert
Burkhead, Jason L.
Burlak, Christopher
Burnett, Arthur
Burnette, Pearlie K.
Burton, Zachary F.
Busse, Björn
Bussolati, Benedetta
Butany, Jagdish
Büttner, Sabrina
Buurman, Ed T.
Buzzini, Pietro
Caballero, Susana
Cabral, Horacio
Cacciola, St. Olga
de Caceres, Dr II
Cacucciolo, Vito
Caffrey, Patrick
Cahill, Patrick L.
Cahoon, A. B.
Cai, Lu
Cai, Weili
Cairrão, Elisa
Caisová, Lenka
Caldeira, Ana Teresa
Call, Douglas R.
Call, Matthew E.
Callister, Steven M.
Calvano, C. D.
Calvert, John W.
Calvisi, Diego F.
Câmara, Niels Olsen Saraiva
Camatini, Marina
Cameron, Barbara
Cammas-Marion, Sandrine
Campagna, Shawn R.
Campbell, Christopher
Campiglia, Pietro
Campos, Carmen A.
Campos, Francisco
Canani, Roberto
Cancello, Raffaella
Candeias, Marco M.
Canini, Antonella
Canonico, Marianne
Canton, Marcella
Cao, Bo
Cao, Cong
Cao, Xiaoyan
Capilla, Encarnación
Caplin, Ben
Carafa, Maria
Caraglia, Michele
Carcangiu, Vincenzo
Cardillo, Carmine
Cardina, John
Cardinali, Daniel P.
Cardon, Ludwig
Cardoso, Luís
Cardoso, Susana M.
Carè, Alessandra
Carew, Anna
Carlin, Kasey Maddock
Carloni, Sante
Carlsen, Monica H.
Carlson, Steve A.
Carlstedt, Thomas
Carlyle, James R.
Carmichael, Gordon G
Carr, John P.
Carrasco, Pedro
Carretero, O. A.
Carrier, Alice
Carrillo-Vico, Antonio
Carvajal, M.
Carvalho, Livia
Carvalho, Pedro
Casamassimi, Amelia
Casanova-Nakayama, Ayako
Casartelli, Morena
Caselli, Chiara
Casida, Mark E.
Casper, Robert F.
Castañeda, Santos
Castan-Laurell, Isabelle
Castelein, René M.
Castell, Alina
Castellano, Gloria
Castellano, José María
Castellino, Robert C.
Castilho, Rogerio M.
Castresana, Carmen
Catara, Vittoria
Cavicchi, Kevin
Cawthorne, Michael A.
Cazorla-Amorós, Diego
Ceccarelli, Sara
Cederberg-Helms, Hans Christian
Cegolon, Luca
Cenci, Elio
Ceolotto, Giulio
Cepeda, Carlos
Ceriotti, Aldo
Cerrada, Maria Luisa
Cervantes, Jorge L.
Cesarino, Igor
Cha, Sangwon
Chaban, Vitaly V.
Chadjichristos, Christos E.
Chai, Yifeng
Chak, Kayam
Chalivendra, Vijay
Chalkley, Robert
Chalmers, Jeffrey J.
Chan, Anthony WH
Chan, Conrad En Zuo
Chan, Elsa C.
Chan, Hsiao Chang
Chan, Juliana C. N.
Chan, Julie
Chan, Koon-Ho
Chan, Ming-Tsair
Chan, Siew Wee
Chan, Wai-Yee
Chand, Hitendra
Chang, Chia-Ching
Chang, Fang-Rong
Chang, Ing-Feng
Chang, John P.
Chang, Lin-Li
Chang, Long-Sen
Chang, Te-Sheng
Chang, Wei-Chiao
Chang, Wen-Chi
Chang, Wen-Wei
Chang, Yu-Jung
Chang, Yung
Chapel, Alain
Chappell, Mark C.
Chapple, Sarah
Charitidis, Costas A.
Charlier, Daniel
Chassande, Olivier
Chassy, Bruce M.
Chatonnet, Arnaud
Chatterjee, Jhinuk
Chaudhary, Kunal
Chemat, Farid
Chemler, Sherry R.
Chemtob, Sylvain
Chen, Bin
Chen, Bing-Hung
Chen, Bor-Kuan
Chen, Chieh-Fu
Chen, Chung-Ming
Chen, Chung-Yi
Chen, Chunjung
Chen, Emily
Chen, George G.
Chen, Guang-Chao
Chen, Guanying
Chen, Guo-Qiang
Chen, Haolin
Chen, Hongzhang
Chen, Huixiong
Chen, Jem-Kun
Chen, Jian
Chen, Jiann-Chu
Chen, Jianye
Chen, Jieli
Chen, Jihua
Chen, Jiun-Rong
Chen, Kun-Song
Chen, Li
Chen, Lingyi
Chen, Po-Yuan
Chen, Qi-Yin
Chen, Rui
Chen, Shu-Hui
Chen, Ssu-Ching
Chen, Tzong-Yueh
Chen, Wei
Chen, Wei-Jan
Chen, Wen
Chen, Wenchi
Chen, X.-B.
Chen, Yang-Yuan
Chen, Yeh-Long
Chen, Yen-Chou
Chen, Yen-Hao
Chen, Ying
Chen, Yingjie
Chen, Yung-Hsiang
Chen, Yun-Wen
Chen, Zhe-Sheng
Chénais, Benoît
Cheng, Alfred S. L.
Cheng, Hua
Cheng, Juei-Tang
Cheng, Kai
Cheng, Kuang-Hung
Cheng, Nikki
Cheng, Ning-Hui
Cheng, Xian Wu
Cheong, JaeHun
Cherian, Philip
Chettimada, Sukrutha
Cheung, K. J.
Cheung, Peter C. K.
Cheung, Po-Yin
Cheung, Wing-Hoi
Chew, Choy-Hoong
Chiang, Han-Sun
Chiang, Tai-An
Chiang, Tzen-Yuh
Chiba, Kazuhiro
Chiesa, Roberto
Chimenti, Sergio
Chiocca, Antonio
Chiono, Valeria
Chiou, Hui-Ling
Chiou, Shih-Hwa
Chitalia, Vipul
Chitalia, Vipul C.
Chlan, Caryl
Cho, Dong-Woo
Cho, Kyoung Sang
Cho, Saeyoull
Cho, Ssang-Goo
Cho, Tae-Joon
Cho, Yangrae
Chobot, Vladimir
Choi, Byong-Seok
Choi, Byung Tae
Choi, Changsun
Choi, Eung H.
Choi, Soo-Jin
Chong, Parkson Lee-Gau
Chou, Chin-Cheng
Chou, James
Chou, Kuo-Chen
Choudhury, Lubna
Choudhury, Swarup Roy
Chow, Wah Soon
Christensen, Lars
Christian, Bartsch
Christmann, Markus
Chrousos, George
Chrubasik, Sigrun
Chrysos, Michael
Chrzanowski, Łukasz
Chu, Ching-Liang
Chu, Fang-Hua
Chu, Yi
Chu, Yiwei
Chuah, Meng Inn
Chuang, Lee-Ming
Chudzinski-Tavassi, Ana Marisa
Chueh, Pin Ju
Chuman, Hiroshi
Chung, Taijoon
Chuu, Chih-Pin
Ciccarelli, Michele
Ciccocioppo, Rachele
Ciccoli, Lucia
Cicero, Arrigo F. G.
Cichero, Elena
Cillessen, Saskia Agm
Cindrova-Davies, T.
Cires, Eduardo
Cirillo, Giuseppe
Ciuman, Raphael Richard
Civetta, Alberto
Clarkson, Andrew
Clawson, Gary A.
Clemetson, Kenneth J.
Clendenen, Tess V.
Cleton-Jansen, Anne-Marie
Cloos, Jacqueline
Cobb, Brian A.
Cobb, John
Cockerill, Gillian W.
Cohney, Solomon
Cohrs, Randall
Coimbra, Eliane Fields
Coleman, Anthony W.
Coleman, Michael
Coleman, William B.
Coley, Helen M.
Coll, Josep
Colliec-Jouault, Sylvia
Collier, Phillip
Collingridge, Graham L.
Collins, Andrew
Colloc'h, Nathalie
Colosimo, Alessia
Comai, Stefano
Conesa-Zamora, Pablo
Constantinescu, Stefan
Conte, Lanfranco
Contestabile, Antonio
Contini, Alessandro
Cookson, S. J.
Cools, Piet
Copani, Agata
Coppini, Raffaele
Coraça-Huber, Débora C.
Corbi, Graziamaria
Corchete, Purificación
Cordewener, Jan H. G.
Cornelissen, Bart
Corominas, M.
Correa-Basurto, Jose
Correia, Sónia C.
Corrigan, Frances
Cortés-Gutiérrez, E. I.
Corthay, A.
Corthesy, Blaise
Corydon, T. J.
Cosic, I.
Costa, Alex
Costa, Carla
Costa, Marta
Costantini, David
Costantini, Federica
Costa-Rodrigues, João
Cotelle, Valérie
Cottee, Nicola S.
Cottet, Martin
Coughlin, Shaun R.
Couillard, Catherine M.
Couldrey, Christine
Coulpier, Muriel
Cowling, Victoria
Crawford, Professor Sybil L.
Crecelius, Anna C.
Crego-Prieto, Victor
Crespo, Mariano Sánchez
Creton, Benoit
Crich, Simonetta Geninatti
Crittenden, Jill R.
Cross, Carroll
Crosthwaite, Susan K.
Cucchiarini, Magali
Cui, Xiaobing
Cullis, Christopher
Culot, Maxime
Cummings, Richard D.
Cummins, Philip M.
Cunha, Angela
Cunningham, Lee Anna
Cunningham, Patrick
Curci, John A.
D. Ritchie, Marylyn
D’hooghe, Matthias
da Cunha, Daniel Andrade
da Silva, M. L. Alves
Dafforn, Katherine A.
Daglia, Maria
Dai, Chi-An
Dai, W.
Dai, Yao
Dai, Yun
Dailey, Michael E.
Dalcin, Daniel
Dall'acqua, Francesco
Dalle, Frederic
Dalmoro, Annalisa
Dalprà, Leda
Dalton, Caroline
Damian, Diona
Damilano, Federico
Damoiseaux, Robert
Daneman, Richard
Daniele, Stéphane
Danielsen, Erik Michael
Danilevskaya, Olga
Dansette, Patrick
Darabi, Hatef
Das, Anindita
Das, Saumya
Das, Undurti
Dasarathy, Srinivasan
Dass, Crispin R.
Dave, Kunjan R.
Davey, Gavin
David Wang, Hui-Min
Davidson, Fordyce A.
Davidson, W. S.
Davie, Elizabeth A. Colby
Davies, Christopher
Davies, Howard V.
Davis, Erica E.
Davis, Randall L.
Davis, Terence
Davletov, Bazbek
Davoli, R.
Dawid, Igor B.
De Angioletti, Maria
De Aza, Piedad N.
De Berardis, Domenico
de Feo, Vincenzo
De Figueiredo, Marcia
De Geest, Bart
De Giglio, Elvira
De Gioia, Luca
De la Mata, F. Javier
De Los Santos, Teresa
De Marco, Iolanda
de Medina, F Sánchez
de Mendonça, Dina I. M. D.
De Michelis, Maria Ida
De Paepe, Boel
de Perrot, Thomas
De Re, Valli
De Riccardis, Federica
De Robertis, Edward
De Rosa, Giuseppe
De Rossi, Anita
De Santa Maria, Luiz Claudio
De Visser, S. P.
de Windt, Leon
Deaglio, Silvia
Dealwis, Chris G.
Dealy, Caroline N.
Dean, Justin
Debode, Frederic
DebRoy, Chitrita
Debyser, Zeger
DeCarlo, Arthur A
Decoursey, Thomas
Deevska, Gergana
Defamie, Norah
Dehouck, Yves
Dekker, Lodewijk
Delcour, Jan A.
DeLeon-Pennell, Kristine Y
Delgado Olguín, Paul
DeMaat, Moniek P. M.
Demontis, Fabio
Demorrow, Sharon
Demyanets, Svitlana
Deng, Lisheng
Denmat, Sylvie Hermann-Le
Denning, Timothy
Dent, Sharon Y. R.
Derkach, K. V.
Desbois, Andrew
Deshpande, Deepak A.
Desouki, Mohamed M.
Dessy, Chantal
Deuchars, Jim
Devaux, Yvan
Devesa, J.
Devos, Damien
Dewi, Mewahyu
Dhar, Sanjit K.
Dharmapatni, Kencana
Dhawan, Deepika
Dhawan, Sangeeta
Dheilly, Nolwenn M.
Dhillon, N. K.
Dhondt-Cordelier, Sandrine
Di Carlo, Marta
Di Giulio, Massimo
Di Maio, Luciano
Di Primio, R.
Di Sansebastiano, Gian Pietro
Di Sotto, Antonella
Di, Rong
Diana, Patrizia
Díaz, José
Díaz-Moreno, Irene
Dickerson, James H.
Dickinson, Gary
Dickinson, Robert
Dieci, Giorgio
Diehl, Phillip
Diekman, Alan
Diekmann, Stephan
Diekwisch, Thomas
Diep, Dzung B.
Dieriks, Birger
Díez-Pascual, Ana
Díez-Tejedor, E.
Digby, Janet
DiGiusto, David
Dijkstra, Bauke W.
Dikalov, Sergey
Dilley, Rodney James
DiMario, Joseph X.
Dimitroulas, Theodoros
Ding, Wen-Xing
Dingermann, Theodor
Dinh, Dzung H.
Dini, Luciana
Dirix, L. Y.
Dixon, David A.
Djordjevic, Julianne
Do, Sun Hee
Dobolyi, Arpád
Dodd, Ian C.
Doig, Andrew
Domingos, Pedro M.
Donaghue, Jacqui
Donatini, B.
Donato, Rosario
Dong, Hong
Dong, Mingdong
Donmez, Gizem
Donnelly, Ryan F.
Donnio, Bertrand
D'Onofrio, Claudio
Dooley, Steven
D'Orazi, Gabriella
D'orazio, John
Dormond, Olivier
Dosio, Franco
Doss, Michael Xavier
Doublet, Patricia
Douroumis, Dionysios
Dracatos, Peter Michael
Drakoulis, Nikolaos
Dreher, Kevin L.
Drobná, Zuzana
Dryer, Stuart E.
Du, William Weidong
Du, Xiao-Jun
Duan, Jianjun
Dübel, Stefan
Ducruet, A. F.
Ducruet, Jean-Marc
Dufès, Christine
Duh, Pin-Der
Duhamel, Jean
Duhamel, Todd
Duivenvoorden, Wilhelmina C. M.
Dunbar, Gary L.
Duong, Tri
Duong-Quy, Sy
Duque, A.
Duque, Paula
Durante, William
Duranti, Marcello
Durruthy-Durruthy, Jens
Durso, Lisa M.
Dutta, Sujoy
Dutton, Susan J.
Duvic, Bernard
Duzgunes, Nejat
Dvir-Ginzberg, Mona
Dvorak, Zdenek
Dyrskjøt, Lars
Dziubla, Thomas
Dzyuba, Sergei
Ebara, mitsuhiro
Ebetino, Hal
Eckman, Delrae M.
Economopoulou, Panagiota
Efron, Philip A.
Eglin, David
Ehrenreich, Hannelore
Ehret, Georg B.
Eichler, Jerry
Eijsink, Vincent
Eisenhut, Marion
Eisenmesser, Elan
Eklund, Britta
Elankumaran, Subbiah
Elder, Alison
Eleftheriou, Eleftherios
El-Essawi, Aschraf
Elhanafi, Ahmad Omar
Elia, Giuliano
Elias, Mikael
Eliseev, Roman A.
Ellington, Andy
Elliott, Steve
Ellsworth, Rachel E.
El-Naggar, Adel K.
Elrouby, Nabil
El-Seedi, Hesham R.
Elsinghorst, Paul W.
Eltom, Sakina E.
Ema, Tadashi
Emaminejad, Sam
Emonard, Hervé
Engeli, Stefan
Engelke, David R.
Englert, Markus
Enninga, Jost
Enns, Gregory M.
Epstein, Murary
Erceg, Slaven
Eriksson, Kristina
Erlinge, David
Eroles, Pilar
Erwin, Patrick M.
Escames, Germaine
Esguerra, Jonathan Lou S.
Eskelinen, Eeva-Liisa
Espinosa-Urgel, Manuel
Estall, Vanessa
Esteban, Maria Ángeles
Esteruelas, Miguel A.
Estevinho, Leticia
Estrada, Vicente
Estrine, Boris
Etheve-Quelquejeu, Mélanie
Etxeberria, Ed
Evans, Janice P.
Evans, Paul
Evans-Galea, Marguerite V.
Exner, Thomas
Fabbri, E.
Fabbri, Francesco
Fabian, Arnberg
Fabio, Vianello
Fabris, Daniele
Fadiel, Ahmed
Fadl, Amin
Fahlman, Richard P.
Fairweather, Delisa
Fåk, Frida
Fan, Liu-Min
Fan, Weimin
Fan, Xiaodan
Fan, Xiucheng
Fanburg, Barry
Fang, Bingliang
Fang, Xianjun
Fantuzzi, Giamila
Farah, Camile
Farahani, Ali Borzabadi
Faraji, Shirin
Faraloni, Cecilia
Farfel, Zvi
Farres, Jaume
Farrow, K. N.
Fassett, Robert
Fassina, Ambrogio
Fathinul, F.
Faul, Christian
Faustino, Amparo
Faustman, Elaine
Fedele, Francesco
Fehling, Heather
Feizi, Soheil
Felsenstein, Kevin M.
Fendri, Imen
Feng, Changjian
Feng, Chia-Hsien
Feng, Xi-Qiao
Feng, Yangbo
Feng, Yibin
Fenn, Michael
Feo, Francesco
Ferlini, Cristiano
Ferlito, Alfio
Fernández, J. A.
Fernández, José J.
Fernandez-Ballester, G.
Fernandez-Botran, Rafael
Fernandez-Garcia, Marta
Fernández-Gómez, Francisco José
Feroci, Marta
Ferrando, M. L.
Ferrarese, Carlo
Ferraresso, Mariano
Ferrari, Paola
Ferrarini, Alberto
Ferraris, S.
Ferreira, A. F.
Ferrières, Vincent
Fessel, Joshua P.
Filichkin, Sergei A.
Filippo, Emanuela
Filleur, Stephanie
Finer, Yoav
Finlay, Graeme J.
Finn, Robert
Finnie, Christine
Fiore, V.
Fiorentino, Alba
Fiorina, Paolo
Firer, Michael
Firth, Andrew
Fischer, Marlene
Fisher, Urs
Fishman, Ayelet
Fishman, Mayer
Fissell, William
Fladung, Matthias
Flahaut, Christophe
Flechsig, Gerd-Uwe
Flieger, Antje
Flisikowski, Krzysztof
Florencio, Francisco J.
Florio, Tullio
Flotats, Albert
Flower, Andrew
Fogolari, Federico
Fokas, Emmanouil
Fomenko, Dmitri E.
Fondevila, Constantino
Fonken, Laura K.
Font, Albert
Foo, Eloise
Ford, Judy
Formisano, Pietro
Fort, Francesca
Fortunato, Orazio
Fossa, Paola
Fossum, Eric
Fosu-Nyarko, John
Fountzilas, G.
Fraga, Braulio M.
Franceschini, Isabelle
Francklyn, Christopher S.
Franco, Catarina
Franco, Pierfrancesco
Franco, Rodrigo
Frank, Patrick
Frattini, Annalisa
Freeman, Michael D.
Freitas, Marisa
Freitas, Michael A.
Freking, Brad
Fresta, Massimo
Friák, Martin
Friedman, Robert
Froeyen, Matheus
Fröhlich, Eleonore
Fröhlich, Holger
Frölich, J.
Fromigué, O.
Frost, Sofia
Frye, Richard E.
Fu, Freddie H.
Fu, Leiping
Fu, Yong-Bi
Lin, Fu-Cheng
Fujimori, Ko
Fujita, Ken-Ichi
Fujita, Masayuki
Fukada, So-Ichiro
Fukao, Takeshi
Fukoura, Hirotaro
Fukuda, N.
Fukuoka, Hiroyuki
Fukushima, Masami
Fukuta, Yoshimichi
Fullston, Tod
Furumatsu, Takayuki
Fuster, Daniel
Gabelli, Sandra B.
Galanakis, Charis M.
Galardy, Paul J.
Galdiero, Stefania
Galeazzi, Roberta
Galiano, Silvia
Galla, Hans-Joachim
Gallardo, Karine
Galli, Andrea
Gallicano, G. Ian
Galván, José Valero
Gambino, G.
Gammone, Maria Alessandra
Gamstedt, E. Kristofer
Gandellini, Paolo
Ganji, Purna Chandra
Gao, Junping
Gao, Shan
Gao, Yonggui
Gara, Naveen
Garau, Gianpiero
García Encina, Pedro A.
Garcia, Antonio
García, Irene
García-Arrarás, Jose E.
García-Jiménez, Custodia
Garcia-Orad, Africa
Gard, Paul
Gardner, Dale
Garg, Parveen K.
Gargiulo, Gaetano
Garinis, George
Garino, Cristiano
Garneau-Tsodikova, Sylvie
Garrisi, V. M.
Garten, Antje
Garzón, Javier
Gasparini, Pierliugi
Gastaldi, Giulia
Gatti, Laura
Gauld, James W.
Gaultier, Alban
Gava, Alessandra
Gavira, José A.
Gazdhar, Amiq
Gazouli, M.
Ge, Shengfang
Ge, Wei
Geeraerd, Annemie H.
Geerts, Hugo
Gehring, Amy
Geilfus, Christoph-Martin
Geirsdottir, Margret
Geitmann, Anja
Gelbart, William M.
Gemei, Marica
Gencoglu, Aytug
Genji, Imokawa
Gentile, Christopher L.
Gentile, Francesco
Gentile, Piergiorgio
Georgakilas, Alexandros G.
Georgiev, Milen
Georgopoulos, Neoklis A.
Gerber, Alexander
Gerchman, Yoram
Gerecke, Kim M.
Gerl, Mathias J.
Gerner, Christopher
Geronikaki, Athina
Geso, Moshi
Gestwicki, Jason E.
Gewirtz, David A.
Ghasemi, Jahan
Ghassemifar, Reza
Ghirardi, Maria
Ghoreschi, Kamran
Giaginis, Costantinos
Giambona, Antonino
Giannattasio, Sergio
Gibert, Yann
Gielen, Stephan
Gilbert, Kathleen
Giles, Thomas D.
Gimnez, Raquel
Giniatullin, R.
Giordano, Antonio
Gironella, Meritxell
Gitto, Rosaria
Gizzo, Salvatore
Glaser, Benjamin
Glatzel, M.
Gleicher, Norbert
Glocker, Erik-Oliver
Glowacki, François
Gnanaguru, Gopalan
Gobe, Glenda C.
Godin, Biana
Goffredi, Shana
Goldberg, Harvey
Golding, Michael C.
Goldring, Mary
Goldstein, Aaron S.
Golestaneh, Nady
Goltsov, Alexey
Gomer, Richard H.
Gomes, Ana P.
Gomez, Ana
Gómez, Ana M.
Gómez, Gustavo
Gomez, Nidia
Gómez-Laguna, J.
Gomez-Llorente, Carolina
Gomez-Quiroz, Luis E.
Gondhalekar, Ameya D
Gondi, Christopher S.
Gondry, Muriel
Gong, Myoung-Seon
Gong, Ping
Gong, Siew-Ging
Gong, Yuewen
Goni, Felix
Gonsalvi, Luca
Gonzales, Bruno
Gonzalez Muniesa, Pedro
González, Víctor M. Víctor
González-Bello, Concepción
Gonzalez-Perez, Ruben R.
Gonzalez-Polo, Rosa A
González-Rodríguez, María Victoria
González-Sarmiento, Rogelio
Gopalakrishnan, Vidya
Gorgoulis, Vassilis G.
Görgün, Güllü
Gorman, Shelley
Gorrell, M. D.
Gosselet, Fabien
Goswami, Prabhat C.
Gotea, Valer
Gott, Jonatha M.
Göttle, Adrien J.
Gough, Albert
Gould, Gwyn
Goulielmos, George N.
Gourion, Benjamin
Gout, Ivan
Govindjee, Govindjee
Gowdy, Kymberly
Goya, Luis
Grabelnych, Olga I.
Grabrucker, Andreas
Gracias, David
Graeber, Kai
Graepel, Rabea
Graf, Norbert
Graham, Brian B.
Graham, Lloyd D.
Graham, Neil
Granberg, Fredrik
Grandfils, C.
Granot, David Granot
Grant, Ross
Grant, William
Graß, Rüdiger
Grassi, Gabriele
Grassi, Giovanni
Grattagliano, Ignazio
Grau, Veronika
Gray, Mark
Gray, N. D.
Greaves, Erin
Greco, Claudio
Greco, Francesco
Greenberger, Joel S.
Greene, Lesley H.
Greene, Nigel
Greenwood, David
Greenwood, Michael T.
Gregory, Christopher
Gregory-Bryson, Emmalena J.
Greim, Helmut
Gresshoff, Peter
Grieco, Carmine R.
Grigorenko, Elena
Gril, Brunilde
Grimaldi, Maurizio
Grimholt, Unni
Grinberg, Daniel
Grisk, Olaf
Grogan, Gideon
Gross, Bradley A.
Gross, Gerhard
Grossini, Dott. E.
Groves, Danja S.
Gruber, Gerald
Gruber, Helen E.
Gruber, Reinhard
Gruenheit, Nicole
Gruzman, A.
Grzmil, Michal
Gu, Wenyi
Gudiminchi, Rama
Gudiña, Eduardo
Guerau-De-Arellano, Mireia
Guerrero, Juan Miguel
Guerriero, Gea
Gugliucci, Alejandro
Guha, Rajarshi
Guillaume-Gentil, Orane
Guillot, Thomas
Guil-Luna, Silvia
Gulati, Pawan
Gulick, Patrick J.
Gullo, Maria
Guo, Jianming
Guo, Min
Guo, Peixuan
Guo, Z. Sheng
Gupta, Ankit
Gupta, Madhulika A.
Gupta, Mukesh Kumar
Gupta, Sanjay
Gupta, Sanjeev
Gurevich, Vsevolod V.
Gust, Kurt A.
Gutiérrez, S.
Gutiérrez-Juárez, Roger
Gutiérrez-Uribe, Janet A.
Gutschner, Tony
Guttery, David
Guttmann, Rodney P.
Guven, Esra Pamukcu
Guzzo, Rosa M.
Ha, Ki-Tae
Habersetzer, François
Hackenberg, Michael
Hadjipavlou-Litina, Dimitra
Haese, Patrick C. D.
Hagedoorn, Peter-Leon
Hagenacker, Tim
Hagenbuch, Bruno
Haglund, Lisbet
Hagmann, Sébastien
Hai, Bo
Haigh, Cathryn
Hajdu, Joseph
Halappanavar, Sabina
Hallberg, B.
Halldén, Sara
Hamanishi, Junzo
Hamberger, Björn
Hamdane, Djemel
Hamik, Anne
Hamrah, P.
Hamza, Adel
Han, Amy
Han, Derick
Han, Jin
Han, Jung Min
Han, Sang-Bae
Han, Sung Soo
Han, Yeon Soo
Han, Yifan
Hanas, Jay
Hancock, Chad R.
Hankinson, Oliver
Hanks, Lynae J.
Hansen, Immo A.
Hanson, Johannes
Hanson, Pamela K.
Haqqi, Tariq M.
Hara, Takafumi
Hardeland, Rudiger
Harder, Juergen
Harding, P.
Hardingham, Jennifer E.
Hardtke-Wolenski, Matthias
Hardy, John George
Hare, Joshua M.
Harmon, John W.
Haroutounian, Serkos A.
Harper, Matthew
Harrison, Paul H. M.
Hart, David A.
Hartmann, Erica
Haselberg, Rob
Hashimi, Saeed
Hashimoto, H.
Hashimoto, Kenji
Hashimoto, Naohiro
Hashimotoa, Takashi
Haspel, Nurit
Hassan, Mohamed
Hatano, Etsuro
Hatano, Tsutomu
Hatta, H.
Hattori, Noboru
Hatzopoulos, Stavros
Haugen, Håvard
Haumaitre, Cécile
Havird, Justin C.
Hayakawa, Tohru
Hayashi, Jun-Ichi
Hayashi, Satomi
Hayashida, Morihiro
Hayes, Maria
Hazlett, Linda D.
He, Jingwei
He, Songbing
Head, David A.
Headley, Allan
Hecht, Jacqueline T.
Hecker, A.
Hedeland, Mikael
Hedley, Peter
Hee, Kang
Hegde, Muralidhar L.
Hehlgans, Stephanie
Heijde, Marc
Heim, Sabine
Heimler, Daniela
Heitz, Thierry
Helariutta, Yrjö
Hellewell, Sarah C
Hellstrom, Ingegerd
Helms, J. Bernd
Helttunen, Kaisa
Hemmer, E.
Hemmerlin, Andréa
Hemmings, D. G.
Hempel, Ute
Henao-Martinez, Andres Felipe
Hendriks, Rudi W.
Hendrix, Mary J. C.
Hennig, Janosch
Henrion, Daniel
Henriques, Romina
Her, Chengtao
Her, Guor Mour
Hermosín-Gutiérrez, Isidro
Hernández Ledesma, Blanca
Hernández, Florencio E.
Hernandez, Nouria
Hernandez-Alcoceba, Ruben
Heron, Dwight
Herrero Ezquerro, Maria-Trinidad
Herrero, María Jesús
Herrmann, Thomas
Hersh, Louis B.
Hershey, John W. B.
Hertog, Jeroen den
Hettich, Robert
Hettinga, Kasper
Heusch, Gerd
Heuzey, Marie-Claude
Hickey, Michael J.
Hidari, Kazuya I. P. J.
Hideyuki, Mitomo
Higashi, Shougo
Higashitani, Atsushi
Higgins, Paul J.
Higo, Junichi
Hinkel, Rabea
Hinsenkamp, Maurice
Hintze, Vera
Hirabara, Sandro M.
Hirai, Itaru
Hirakawa, Kazutaka
Hiramoto, Keiichi
Hirano, Katsuya
Hirase, Hajime
Hirashima, Akinori
Hirata, Yoshihiro
Hird, Michael
Hiroaki, Fujii
Hiroaki, Ooboshi
Hirota, Kiichi
Hiroto, Satoru
Hirsch, Michelle S.
Ho, Michael (Shan-Hui)
Ho, Shin-Lon
Ho, Wen-Fu
Ho, Yuan-Soon
Hocevar, Barbara A.
Hochreiter, Sepp
Hocquette, Jean-François
Hodson, David J.
Hoffman, Robert
Hoffmann, Georg
Hoffmann, Katherine
Hoffmann, Thomas J.
Höfinger, Siegfried
Hogarth, Cathryn A.
Hogberg, Helena
Hogg, Philip
Holen, Ingunn
Holinstat, Michael
Holland, William L.
Holloway, Alison C.
Holmøy, Trygve
Holstein, G. R.
Holtzman, Jordan L.
Homer, Hayden Anthony
Honda, Kazuhisa
Hondermarck, Hubert
Hong, Jin Tae
Hong, Jongki
Hong, Kyung U.
Hong, Yonggeun
Hong-Brown, Ly Q.
Honoré, Patrick M.
Hoole, Stephen P.
Hooven, Louisa
Hooykaas, Paul
Hori, Tomohide
Hörkkö, Sohvi
Horn, Simon
Hornberger, Troy A.
Horner, Ashley
Horwood, Nicole J.
Hosgood III, H. Dean
Hostager, Bruce
Hotta, Mikinori
Hotz, Nico
Hou, Anfu
Hou, Chien-Wei
Hou, Tingjun
Houen, Gunnar
Hour, Mann-Jen
Houston, Ross
Howes, Melanie
Howie, Sarah
Howitt, Jason
Hricak, Hedvig
Hsieh, Chi-Hsun
Hsieh, Yih-Shou
Hsieh, Yi-Hsien
Hsing, Chung-Hsi
Hsu, Cheng-Che
Hsu, Chin-Lin
Hsu, Hsiu-Fu
Hsu, Li-Sung
Hsu, Ming-Jen
Hsu, Todd
Hsueh, Yu-Mei
Hu, Che-Ming Jack
Hu, Fenghua
Hu, Jer-Ming
Hu, Lingzhi
Hu, Ming-Chang
Hu, MingKuan
Hu, Tianhui
Hu, Valerie W.
Hu, Xiaoming
Hu, Zhuowei
Huang, A-Mei
Huang, Bu-Miin
Huang, Chiu-Jung
Huang, Chi-Yu
Huang, Jian
Huang, Jianying
Huang, Ming Te
Huang, Tai-Chung
Huang, Tao
Huang, Tien L.
Huang, Wei-Chien
Huang, Wen-Lii
Huang, Ya-Ling
Huang, Ying
Huang, Ying-Hsien
Huang, Yu
Huefner, Antje
Huen, Sarah C.
Hull, Joe
Humblot, Vincent
Humpel, Christian
Hund, Thomas J.
Hung, Andrew
Hung, Chao-Hung
Hung, Jan-Jong
Hupp, Ted
Huppi, Konrad
Hur, Mina
Hurdle, Julian
Hurel, Charlotte
Hurlé, Juan
Hurst, Robert E.
Hurst, Roger D.
Husa, Matt
Husain, Mainul
Hussain, Saber M.
Hussain, Salik
Hvidsten, Torgeir R.
Hwang, Daniel H.
Hwang, Jeng-Jong
Hwang, Tsong-Long
Hyogo, Hideyuki
Hyun, Sang-Hwan
Iacobazzi, Vito
Iafisco, Michele
Ibiebele, Torukiri I.
Ichihara, S.
Idoyaga, Juliana
Igwe, Orisa J.
Iinuma, Hisae
Iles, Ray
Im, Chaeuk
Im, Hee-Jeong
Imamura, Masanori
Imamura, Tetsuya
Imhof, Petra
Inaba, Kenji
Inácio, Angela
Incoronato, Mariarosaria
Inestrosa, Nibaldo C.
Inman, Sharon
Inoue, Atsuko
Inoue, Ken-Ichiro
Inoue, Naoya
Insam, Heribert
Insel, Paul A.
Inukai, Yoshiaki
Inuzuka, Hiroyuki
Invernizzi, Pietro
Iorizzi, Maria
Iqbal, Samir
Irache, Jose Manuel
Iraci, Nunzio
Irato, Paola
Irwin, David C.
Isaka, Yoshitaka
Ishido, Masami
Ishii, Isao
Ishii, Tetsuro
Ishikawa, Haruki
Ishikura, Minoru
Ishimaru, Kanji
Ishino, Fumitoshi
Ishizaki, Takuma
Ishmael, Faoud T.
Ishman, P. F.
Ismail, Heba M. S.
Isobe, N.
Isoda, Katsuhiro
Isokpehi, Raphael D.
Istvan, Boldogh
Ito, Akihiko
Ito, Michiho
Ito, Shigeki
Itoh, Ken
Itskov, Mikhail
Iuchi, T.
Ivell, Richard
Iwaki, Takayuki
Iwamori, Masao
Iwamoto, Ryo
Iwamoto, Sadahiko
Iwaniec, Urszula T.
Iwasaki, Kenta
Iwasaki, Tomohiro
Iwasaki, Yugo
Iwata, Shigeru
Izabel Camargo, Maria
Izumi, N.
Izumi, Yasuo
Jabbari, Siavash
Jaber, Maguy
Jablonska, Ewa
Jacob, Francis
Jacobsen, Donald W.
Jaeger, Henry
Jaeger, Kinan
Jaeschke, Hartmut
Jahandideh, Samad
Jaime, Laura
Jain, Abhinav
Jain, Mohan Shri
Jakobsdottir, Greta
Jalili, Thunder
Jalowiecki-Duhamel, L.
James, Philip
Janczarek, Monika
Janda, Elzbieta
Janfelt, Christian
Jang, Byoung Kuk
Jang, Kyung Lib
Jang, Seonghoe
Janin, Joël
Janji, Bassam
Janouškovec, Jan
Jansen, Gerrit
Jarmuszkiewicz, Wieslawa
Jarret, Robert L.
Jat, Parmjit S.
Jaudal, Mauren Jaudal
Javanmard, Mehdi
Javier Rodríguez, Francisco
Jaworska, Natalia
Jayasinghe, Suwan
Jeanplong, Ferenc
Jedlinek, Herbert
Jeeninga, Rienk E
Jelinek, D. F.
Jennings, Jessica A
Jennings, Robert M.
Jeon, You-Jin
Jeong, Ki Jun
Jeschke, Marc
Jeschke, Udo
Jessop, Forrest
Jesus Raposo, Maria Filomena
Jeyaseelan, Kandiah
Ji, Junyuan
Jia, Chenxi
Jia, Fan
Jia, Qi
Jiang, Liwen
Jiang, P. X.
Jiang, Shu-Ye
Jiang, Xian
Jiang, Xiaohua
Jimeno, Ciril
Jin, Chongwei
Jin, Guangfu
Jin, Sha
Jin, Yan
Joanne, Yip
John, James A. St.
Johnsen, John Inge
Johnson, Kristen
Johnson, Randy L.
Johnson, Tom
Joles, Jaap A.
Joly, David
Jonas, Jean-Christophe
Jones, Jessica L.
Jones, K.
Jones, Kevin B.
Jonesa, Alan M.
Jonesa, G. T.
Joo, Hong-Gu
Jose M, Alía
Josefsson, Emma
Joseph, Winaver
Joshi, Mandar S.
Joven, Jorge
Jovin, Thomas
Julian, Colleen Glyde
Jung, Klaus
Jung, Seunho
Jung, Youngmi
K. Deyholos, Michael
Kaczor, Agnieszka
Kadota, Kazunori
Kadowaki, Norimitsu
Kagadis, George C.
Kagawa, Tetsushi
Kahaly, G. J.
Kahle, Philip P.
Kaina, Bernd
Kaji, Hirokazu
Kajiya, Kentaro
Kakar, Sham
Kako, Tetsuya
Kalajdzic, Predrag
Kalamarides, M
Kaludercic, Nina
Kambe, Naotomo
Kamiya, Hidehiro
Kämmerer, Peer W.
Kanaoka, Masahiro
Kanda, Tatsuo
Kandimalla, Ekambar R.
Kandola, Baljinder
Kane, Patricia M.
Kaneko, Kazunari
Kaneoka, Hidenori
Kang, Byoung-Cheorl
Kang, H.
Kang, Hunseung
Kang, Kwon-Kyoo
Kang, Kyung Pyo
Kang, Se Chan
Kang, Sinyoung
Kanno, Yuichiro
Kantartzis, Konstantinos
Kantharidis, Phillip
Kanzaki, Hiroyuki
Kao, Jia-Horng
Kapiloff, Michael S.
Kapoor, Mohit
Karabencheva, Tatyana
Karakasidis, T. E.
Kärenlampi, Sirpa
Karger, Axel
Karginov, Ted
Karlic, Heidrun
Karpukhina, Natalia
Kaschina, Elena
Kase, Satoru
Kashiwakura, Ikuo
Kashyap, Rahul
Kasimanickam, Vanmathy R.
Kasiotis, Konstantinos M
Kassiri, Zamaneh
Kataoka, Hiromi
Katayama, Shigeru
Katinakis, Panagiotis
Kato, Hirohito
Kato, Tadafumi
Kato, Tatsuya
Kato, Yoko
Katoh, Masaru
Katrin, Becker-Flegler
Katsel, Pavel
Katsuki, Hiroshi
Katsuta, Shoichi
Kaur, Charanjit
Kaur, Gurbinder
Kav, Nat N. V.
Kawaguchi, H.
Kawai, Toshiyuki
Kawano, Tsuyoshi
Kawasaki, Hideki
Kawazu, K.
Kayano, Shin-Ichi
Kayser, Oliver
Keeble, Julie Elizabeth
Kefas, Benjamin
Kelleher, Fergal C.
Kemp, Kevin
Keng, Vincent W.
Keong, Kwoh Chee
Kern, Wolfgang
Kersten, Sander
Kessel, David
Kessler, Vadim
Kessler-Icekson, Gania
Keul, Helmut
Khalaila, Isam
Khamisy, Sherif F. El
Khan, Anzar
Khanna, Savita
Khurana, Sandeep
Kiani, Mohammad F.
Kichambare, Padmakar D.
Kidd, La Creis R.
Kidron, Heidi
Kieda, Claudine
Kiesslich, Tobias
Kihara, Nobuhiro
Kikuchi, E.
Kim, Albert H.
Kim, Beom Joon
Kim, Chong Jai
Kim, Deok Ryong
Kim, Gyoo Cheon
Kim, Hak Yong
Kim, Hyun-Woo
Kim, Ick Young
Kim, Il-Man
Kim, Jae Bum
Kim, Jae Kwang
Kim, Jae-Hong
Kim, Jayoung
Kim, Jeong Hun
Kim, Jin Cheon
Kim, Jin-Wook
Kim, Ji-Su
Kim, Jong Sung
Kim, Jong-Min
Kim, Kiyoung
Kim, Kwang S.
Kim, Kwang Sik
Kim, Min Young
Kim, Ok Tae
Kim, S.
Kim, Sang-We
Kim, Seong-Gon
Kim, Sun Hwa
Kim, Sun Yeou
Kim, Sung Hoon
Kim, Sung Soo
Kim, Sungjee
Kim, Tae Hoon
Kim, W. K.
Kim, Won Ho
Kim, Woo Ho
Kim, Yong-Dae
Kim, Yong-June
Kim, Young-Mok
Kim, Young-Sik
Kim, Youngsoo
Kind, Tobias
Kindy, Mark
King, Paul W.
Kinsel, Gary
Kinsey, William H.
Kinugawa, S.
Kipp, Anna P.
Kirby, Ralph
Kirchhoff, Frank
Kirimura, Kohtaro
Kirk, Martin L.
Kirsch, Gilbert
Kiselyov, Kirill
Kitagawa, Fumihiko
Kitajima, Shigetaka
Klampfer, Lidija
Klatt, Christian G
Klaver, Caroline
Klechevsky, Eynav
Klegeris, Andis
Klein, David C.
Kleindienst, Andrea
Kleszczyński, Konrad
Kleuser, Burkhard
Klinke, David
Kloc, Malgorzata
Klokov, Dmitry Y.
Klugbauer, Norbert
Knapp, Stefan
Knetsch, Menno L. W.
Knippschild, Uwe
Knox, Paul
Knüppel, Sven
Ko, Hyun-Jeong
Ko, Jane L.
Ko, Jih-Yang
Ko, Jiunn-Liang
Ko, Young Tag
Ko, Young-Gyu
Kobayashi, H.
Kobayashi, Masato
Kobayashi, Yoshiro
Kobayashi, Yukiho
Kobeissy, Firas H.
Koblmüller, Stephan
Köcher, Thomas
Köck, Robin
Koebnik, Ralf
Koek, Ger H.
Koga, Fumitaka
Koh, Young Ho
Koh, Young-Hag
Kojima, Akiko
Kojima, Chie
Kojima, Masami
Kojima, Tetsuya
Kok, Jan
Kok, Klaas
Kokoh, K. B.
Kolbe, L.
Kolehmainen, Erkki
Kolettis, Theofilos M.
Kolovou, Genovefa
Kolppo, Kari
Komasa, Satoshi
Komine, Mayumi
Kondo, Eisaku
Kondo, Hidehiro
Kondo, Tadashi
Kondratov, Roman V.
Kong, Yawei
Konje, Justin
Konsman, Jan-Pieter
Kontro, Inkeri
Koo, In Sun
Koole, Leo H.
Koos, Antal A.
Kooyman, David L.
Kopra, Kari
Koraishy, Farrukh M.
Korsching, Eberhard
Kossida, Sophia
Kösters, Markus
Kostiainen, Mauri A.
Kostopoulos, Spiros
Kötting, Carsten
Kourkoumelis, Nikolaos
Koves, Tim
Kowalczyk, Mariusz
Koya, Daisuke
Koyama, Hiroyuki
Kramer, Phillip
Krantz, Bryan A.
Kratzer, Regina
Kraus, Virginia B.
Krawinkel, Michael B.
Krenek, Sascha
Kretz, Markus
Kreutz, Rolf P.
Kristian, Tibor
Kroner, E.
Kronholm, Ilkka
Krysko, Olga
Kuan, Yu-Hsiang
Kubitscheck, Ulrich
Kuboki, Takuo
Kubota, Naoto
Kudo, Yasusei
Kues, Wilfried A.
Kukol, Andreas
Kuliawat, Regina
Kulka, Marianna
Kumar-Singh, Samir
Kundu, Joydeb Kumar
Kuntz, Marcel
Kunze, Gotthard
Kuo, Mei-Ling
Kuo, Tzong-Fu
Kuramae, Eiko
Kurasaki, Masaaki
Kurdowska, Anna K.
Kurgan, Lukasz A.
Kurhanewicz, John
Kurita, Hirofumi
Kurokawa, Masahiko
Kurose, Hitoshi
Kurreck, Jens
Kurtti, Timothy
Kusakabe, Takahiro
Kusano, Miyako
Kuschal, Christiane
Kushibiki, Toshihiro
Kuster, Diederik
Kusumoto, Norihisa
Kuwahara, Koichiro
Kvítek, Libor
Kweon, Oh-Kyeong
Kwon, Do Hoon
Kwon, Dong-Yeul
Kyprianou, Natasha
Kypta, Robert
L Budarin, Vitaliy
Laakkonen, Pirjo M.
Laane, R. W. P. M.
Lacaille-Dubois, Marie-Aleth
Lacerra, Giuseppina
Ladeira, Carina
Laederach, Alain
Laekeman, Gert
Lagrost, Corinne
Laguna, Mariano
Laher, Ismail
Lahiri, Abhishek
Lai, Chien-Chen
Lai, Hsin-Chih
Lai, Xianyin
Lakin-Thomas, Patricia L.
Lal, Shailesh K.
Lalowski, Maciej
Lam, Hon Ming
Lam, Jenny K. W.
Lamartine, Jérôme
Lambrides, Christopher J.
Lanaspa, Miguel A.
Landis-Piwowar, Kristin
Landreville, Solange
Lane, Lydie
Lanekoff, Ingela
Lang, Siegmund
Langel, Walter
Langner, Cord
Lanteri, S.
Lapierre, Pascal
Lapres, John J.
Laranjo, Marta
Larizza, Lidia
Larjava, Hannu S.
Larkin, Jonathan
Larkin, Philip J.
Larrainzar, Estibaliz
Larsen, Søren T.
Larson, Nicholas B.
Larsson, Per
Lartigue-Faustin, Lydia
Lash, Larry
László, Juhász
Lathia, Justin
Lathrop, Richard H.
Lau, Hang Yung Alaster
Lau, Joseph
Lau, Kin-Hing William
Lau-Cam, Cesar A.
Lauria, Antonino
Lauterbach, E. C.
Lauzon, Carol R.
Lavandera, Ivan
Lavelli, Vera
Lawless, Matthew W.
Lazartigues, Eric
Lazzara, Giuseppe
Lazzarato, Loretta
Le Borne, Sylvie
Le Visage, Catherine
Le, Quang Si
Leach, Katie
Leal, Ermelindo C.
Ledesma, Maria Dolores
Lee, Bor-Shiunn
Lee, Byong
Lee, C.-H.
Lee, Changjoon Justin
Lee, Chang-Soo
Lee, Ching-Kuo
Lee, Chong-Kil
Lee, Dae Ho
Lee, Davy Cw
Lee, Dong Hoon
Lee, Eunjoo H.
Lee, Eun-Ok
Lee, Hangil
Lee, Hsinyu
Lee, Huei
Lee, Hye Suk
Lee, Hyunjoo
Lee, In Seop
Lee, J.-H.
Lee, Jae Hoon
Lee, Jae Hwan
Lee, Jae-Ho
Lee, James C.
Lee, Jandee
Lee, Jeong-Chae
Lee, Jin Soo
Lee, Jong-Hwan
Lee, Jun Sik
Lee, Ki Hoon
Lee, Kwang Ho
Lee, Kyu Wan
Lee, Lin-Wen
Lee, L. T. O.
Lee, Myung Koo
Lee, Sanghyeob
Lee, Taeseung
Lee, Tzong-Shyuan
Lee, Vincent Ws
Lee, Wei
Lee, Wen-Ying
Lee, Wing-Kee
Lee, Wonhye
Lee, Wonoh
Lee, Woo Sung
Lee, Yang Won
Lee, Yeon-Ju
Lee, Yeon-Su
Lee, Yong Sun
Lee, Young
Lee, Young Ho
Lee, YoungJoo
Lee, Yun-Shien
Lee, Zang Hee
Leegwater, P. A. J.
Legault, Pascale
Lehtonen, Sanna
Lei, X.-H.
Lei, Xia
Leicht, Anthony
Leifert, Wayne R
Lellouche, Nicolas
Lemarié, Anthony
Lemasson, Isabelle
Lenaz, Giorgio
Lenzen, Sigurd
Leonard, Alan C.
Leonard, Anna V.
Leonardi, Salvatore
Leong, David Tai
Leoni, Giovanni
Leonidas, Demetres D.
Leopold, Jane A.
Lerch, M.
Lesch, Bluma J.
Leslie, J. C.
Lessard, Christopher
Lessman, Charles
Leszczynska, D.
Leto, Thomas L.
Leu, Steve
Leu, Sy-Jye
Leung, Angela M.
Leung, Patrick S. C.
Levine, Beth
Levinson, Ralph D
Levkau, Bodo
Levy, Oren
Lewis, Sheena E. M.
Lewy, Alfred J.
Ley, A. C.
Lezot, Frédéric
Li, Albert P.
Li, Ang
Li, Bailing
Li, Bing
Li, Chi
Li, Dongpei
Li, Fengzhi
Li, Gang
Li, George
Li, James C. B.
Li, Jingjing
Li, Jinjun
Li, Jinping
Li, Kui
Li, Kuo-Bin
Li, Maoyin
Li, Menglong
Li, Ning
Li, Qingshun
Li, Rongpeng
Li, Rugang
Li, Shaoping
Li, Tiangang
Li, Tieshi
Li, Tsung-Lin
Li, Wen-Hong
Li, Xiang-An
Li, Xiaohui
Li, Xudong Joshua
Li, Yi
Li, Yinxin
Li, Yongfang
Li, Yuexian
Li, Zonghai
Liang, Carol Jiurong
Liang, Chia-Hua
Liang, Gaolin
Liang, Jie
Liang, Li
Liang, Po‑Huang
Liang, Xue-Hai
Lianidou, Evi
Liao, Chien-Sen
Liao, Joshua
Liao, Pei-Chun
Liao, Ying-Chih
Libert, Sergiy
Liebau, Stefan
Lilja, Mirjam
Lim, Keng Gat
Lim, Kyung-Min
Lim, Sung-Jig
Lim, Yoongho
Limpens, Erik
Lin, Chang-Shen
Lin, Chao-Nan
Lin, Chih-Chien
Lin, Chih-Yuan
Lin, Chii-Wann
Lin, Chiou-Feng
Lin, Guiting
Lin, Gui-Ting
Lin, Hai-Shu
Lin, Ho
Lin, Honghuang
Lin, Huilan
Lin, Hung Wen
Lin, Hung-Yin
Lin, Jin-Yuarn
Lin, Junyan
Lin, K.-H.
Lin, Ming-Chung
Lin, Nancy
Lin, Qinsong
Lin, Shengda
Lin, Shu
Lin, Shwu-Jiuan
Lin, Tsan-Piao
Lin, Wan-Wan
Lin, Wensheng
Lin, Xi
Lin, Yenshou
Lin, Ying-Ju
Lin, Yu-Hsin
Lin, Yun-Lian
Linard, Christine
Lindeman, Jan
Lindermayr, Christian
Lindfors, N. C.
Lindholm, Dan
Lindsay, Denise
Lindstrand, Anna
Lindström, Mikael
Linert, Wolfgang
Ling, Geoff
Ling, Hui
Ling, Maurice Ht
Ling, Yong-Chien
Linnebacher, Michael
Linsell, Katherine
Liou, Jing-Ping
Liou, Ying-Ming
Lippens, Guy
Lippitz, Bodo
Lirk, P.
Lisman, Ton
Lisse, Thomas
Little, Peter J.
Liu, Allen P.
Liu, Alvin Y.
Liu, Bin
Liu, Chia-Yang
Liu, Chun-Hung
Liu, Delong
Liu, Hung J.
Liu, Jiandong
Liu, Jie
Liu, Li
Liu, Li-Feng
Liu, Ming
Liu, Ping
Liu, Qiang
Liu, Renyi
Liu, Shing-Hwa
Liu, Shiuh-Tzung
Liu, Song
Liu, Song-Tao
Liu, Tie Fu
Liu, Tzong-Shi
Liu, Xiaohua
Liu, Xuehong
Liu, Xueqian
Liu, Yonghong
Liu, Zhandong
Lizarraga, Karlo J.
Lleò, Maria M.
Lo, Huang-Mu
Lo, Jeng-Fan
Lo, Kevin W.-H.
Lo, Mindy S.
Lo, Peggy P. K.
Lo, Pui-Chi
Loarca-Piña, Guadalupe-Flavia
Loboda, Agnieszka
Lockridge, Oksana
Lodola, Alessio
Loeb, David M.
Loffredo, Lorenzo
Lofrumento, Dario D.
Loh, Xian Jun
Lohi, Olli
Loike, John
Lokshin, Anna
L'Ollivier, Coralie
Lombard, David B.
Lonardo, Amedeo
Lopes, F.
López, Adrián González-
López, F.
Lopez, Mandi
López-Jaramillo, F Javier
Loprinzi, Charles L.
Lord, Edith
Lorenz, Kristina
Lorenzetti, Diego
Lorenzo, O.
Lorite, I.
Losano, Gianni
Louime, Clifford
Louis, Petra
Louria-Hayon, Igal
Lowe, Martin
Lpez-Legentil, Susanna
Lu, Rui Ray
Lu, Binfeng
Lu, Hong
Lu, Kuang-Hui
Lu, Lianghao
Lu, Ming-Wei
Lu, Pei-Luen
Lu, Tse-Min
Lu, Xiaonan
Lu, Ze
Lübeck, Mette
Lucas, Christopher D.
Luco, Reini F.
Ludwig, Marie-Gabrielle
Lugnier, Claire
Lui, Vincent Chi-Hang
Luijsterburg, Martijn
Lum, Julian J.
Lumini, Alessandra
Lundqvist, Andreas
Lundstrom, Kenneth
Luo, Feng
Luo, Xuelian
Lupiáñez, José A.
Lustig, Robert
Lütteke, Thomas
Lutter, Petra
Lynch, Cian J.
Lysaght, J.
Ma, Chao-Mei
Ma, Edmond Dik-Lung
Ma, Guojia
Ma, Hong
Ma, Qiang
Ma, Xiaosong
Ma, Xiaoyue
Ma, Yi
Ma, Yongjie
Ma, Yuelong
Maak, S.
Maass, Alexander H.
Macchiarini, Paolo
MacDonald, Justin A.
MacDonald, Ruth
Mace, Thomas
Machado, Artur da Câmara
Machelon, Véronique
Macias, Rocio I. R.
Mackintosh, Carol
Mackman, Nigel
Macossay, Javier
Madduri, Srinivas
Madej, Thomas
Madesis, Panagiotis
Madhukar, Burra
Madry, Henning
Maeda, Kazuhiko
Maeda, Kazuya
Maeda, Shiro
Mäenpää, Johanna U.
Maestri, Elena
Magiatis, Prokopios
Maglia, Giovanni
Magliano, Dianna J.
Magor, Katharine E.
Mahasenan, Kiran
Maher, William
Mahimainathan, Lenin
Mahony, Timothy John
Mai, Antonello
Maillard, Ludovic
Maki, Kosuke
Malaguarnera, Michele
Malemud, Charles J.
Mallanna, Sunil K.
Mallepally, Rajendar R.
Malling, Tine
Maly, Kenneth E.
Mamo, Gashaw
Manara, Anna
Mancheño, José
Manchia, Mirko
Mandrich, Luigi
Mangoni, Arduino A.
Manian, Avinash P.
Maniwa, Yoshimasa
Mann, Francis
Mannello, Ferdinando
Mannen, H.
Mano, João F.
Manolson, Morris F.
Manoranjan, Branavan
Mansoorabadi, Steve
Mantha, Anil K.
Manzanares, Paloma
Maojo, Victor
Mapelli, Marina
Maraldi, Tullia
Marcelli, Augusto
Marcello, Alessandro
Marchetti, Philippe
Marchiò, Caterina
Marcos, Mercedes
Marcotullio, Maria Carla
Marcu, Kenneth B.
Marcucci, Fabrizio
Maréchal-Drouard, Laurence
Maria-Consuelo del Cañizo, M. C.
Mariani, Giuliano
Mariggiò, Maria A.
Marin-Castaño, Maria E.
Marini, Juan C.
Marinò, Michele
Marino, Silvia
mario, dellagli
Mariottini, Gian Luigi
Markwardt, Fritz
Marmiroli, Nelson
Maroteaux, Luc
Marques, Fernanda
Marriott, Ian
Marsano, Francesco
Marsolais, Frédéric
Martel, Sophie
Martens, John Wm
Martin, Harry
Martin, Luc J.
Martín, María Ángeles
Martin, Melanie
Martin, R. A.
Martin, Sarah A.
Martin, Seth S.
Martin, T. J.
Martin-Diana, Ana Belen
Martinelli, Adriano
Martinez, Alexandre
Martinez, Javier
Martinez, Manuel
Martínez-Abarca, Francisco
Martínez-Chantar, Maria L.
Martinez-Marcos, Alino
Martino, Sabata
Martins, Tiago
Martos Moreno, Gabriel Angel
Marty, Jean-Daniel
Martynyuk, Anatoly E.
Maruyama, Ichiro N.
Maruyama, Kei
Maruyama, Toru
Masaki, Takayuki
Masci, Stefania
Masilamani, Madhan
Masotti, Andrea
Màsson, Màr
Masters, Colin L.
Mastrangelo, Anna M.
Masuda, Haruchika
Masuda, Yuki
Mataftsi, A.
Mateo, Estibaliz
Matés, José M.
Matilla, A. J.
Matsopoulos, George K.
Matsuda, Hisashi
Matsuda, Yoko
Matsueda, Satoko
Matsugo, Seiichi
Matsui, Hirotaka
Matsumiya, Masahiko
Matsumoto, Akikazu
Matsumoto, Kozo
Matsumoto, Takayuki
Matsumura, Sachiko
Matsumura, Takeshi
Matsuoka, Daisuke
Matsuoka, Masaaki
Matsushita, Takehiko
Matsuura, Bunzo
Matsuya, Yuji
Mattarelli, Paola
Mattei, Fabrizio
Mattey, Derek L.
Matthew Ng, Chau Hsien
Matthews, Jason
Mattoo, Abid R.
Matusiewicz, Magdalena
Mavragani, Clio P.
May, Petra
Mayer, Andreas
Mayer-Miebach, Esther
Mazière, Cécile
Mazza, Tommaso
Mazzoccoli, Gianluigi
Mcainsh, Martin R.
McBain, Andrew
McCarty, Owen
McCormick, Alison
Mcdonald, Kerrie
Mcdonald, Peter
Mcdougal, Owen M.
Mcdougall, G. J.
McFeeters, Robert L.
McGill, Mitch
Mcginley, John
Mcinnes, Campbell
McIntosh, Marla S.
Mcmorrow, Tara
Mcnamara, Shamus
Meachem, Sarah J.
Meade, Kieran
Medeiros, Rui
Medema, Jan Paul
Medina, Francisco Javier
Mehlig, Kirsten
Mehra, Reena
Mehta, Ashish
Mehta, Bijal K.
Mei, Nan
Meins, Jean-François Le
Meiracker, Anton H. Van Den
Melkani, G. C.
Melloni, Edon
Memboeuf, Antony
Memelink, Johan
Menendez, Jose Carlos
Meng, Fanhong
Meng, Huan
Meng, Ling
Menna, Marialuisa
Menon, Madhav
Ménoret, Séverine
Menten, Bjorn
Mercader, R. J.
Mercer, Jason
Mercier, Y.
Merino, Gracia
Merkler, Doron
Merriman, Tony R.
Meruvu, Sunitha
Meyer, Rachel
Meyer-Hoffert, Ulf
Meyerholz, David
Mezzanzanica, Delia
Mi, Huaiyu
Mi, Qing-Sheng
Michael, Iba
Michel, Martin
Michelsen, Kathrin S.
Michiels, C.
Michiels, Carine
Michielsen, Peter
Midde, K.
Mielczarek, C.
Mieog, Jos C.
Migliaccio, Anna Rita
Migliaresi, Claudio
Miguel, M. G.
Mikami, Toshinari
Miki, Yasuhiro
Mikkelsen, Deirdre
Mikoshiba, Katsuhiko
Milan, Z.
Milcarek, Christine
Millard, Tom H.
Millecamps, Magali
Miller, Aaron W.
Miller, Allison
Miller, Barbara A.
Miller, Dylan V.
Miller, E.
Miller, Ingrid
Miller, John
Miller, Vandana
Milne, Kevin
Milner, Caroline M.
Minamino, Tohru
Minas, Kostas
Minelli, Alba
Minutolo, Filippo
Miosge, Nicolai
Mir, Mustafa
Miranda, Carlos
Mirande, Marc
Miron, Richard J.
Mirzaei, Mehdi
Mirzayans, Razmik
Misawa, Kazuharu
Misery, Laurent
Mishra, Amaresh
Mishur, Robert J.
Mita, Giovanni
Mitchell, Jennifer A.
Mithöfer, Axel
Mitra, Alok K.
Mitra, Ashim K.
Miyamoto, Masaaki
Miyamoto, Takeshi
Miyaoka, Hiroaki
Miyashita, Tomoyuki
Miyatake, Shin-Ichi
Miyoshi, Taro
Mizuno, Hideaki
Mizuno, Tetsuya
Mo, Huanbiao
Mocanu, G.
Modena, Piergiorgio
Modugno, Francesca
Moeslinger, Thomas
Mognato, Maddalena
Mohabatka, Hassan
Mohan, Helen M.
Mohanty, Debasish
Moldovan, Florina
Moll, Frans
Molvarec, Attila
Monaco, Edward A. III
Monard, Gerald
Mondragon, Alfonso
Moniche, F.
Monji, Akira
Monteiro Ramalhinho, Ana Cristina
Montenarh, Mathias
Monti, Maria Chiara
Moore, Paul H.
Moore, Tara C. B.
Morales, Rodrigo
Morán, José M.
Morandi, Luca
Morchón, Rodrigo
Moreno, J. J.
Moretó, Miquel
Morgan, Richard
Morgan, William F.
Mori, Kiyoshi
Moriggl, Richard
Moriguchi, Shigeki
Morikawa, Masaaki
Morioka, Norimitsu
Morita, Kyoji
Moriya, Takahiro
Mörl, Mario
Morris, Brian J.
Morris, Craig F.
Morris, John
Morris, Viola B.
Mort, John S.
Morton, Charles Oliver
Moscardó, Antonio
Moschetta, Michele
Moseke, Claus
Moshou, Dimitrios
Mostafa, El-Sayed
Mostaghel, Elahe A.
Mota, Manuel
Motoi, Yumiko
Mott, Justin L.
Motterlini, Roberto
Mottet, D.
Mourier, Arnaud
Moussian, Bernard
Mowat, Christopher G.
Mroginski, Maria Andrea
Mruk, Dolores D.
Mu, Qingxin
Mu, Ting-Wei
Mücke, Thomas
Mueller, Wolf C.
Mueller-Roeber, Bernd
Mukaida, Naofumi
Mulcahy-Levy, Jean
Muleo, Rosario
Müller, Marius N.
Muller, Patricia A. J.
Mulvihill, Erin
Mundy, D. C.
Munekazu, Yamakuchi
Muñiz, Manuel
Muñoz-Hoyos, Antonio
Murafuji, Toshihiro
Murai, Koji
Murakami, Masaaki
Murakami, Yasufumi
Murakami, Yoshiki
Muramatsu, Masaaki
Murashita, Koji
Murata, Yoshiyuki
Murate, Takashi
Muro, Andres
Murono, Shigeyuki
Muroyama, Akiko
Murphy, E. Angela
Murtas, Daniela
Murtola, Teemu J.
Musio, Antonio
Mustafa, S. Jamal
Musumeci, Giuseppe
Muzard, Julien
Näär, Anders M.
Naciri, Yamama
Nadeau, Nicola J.
Nagano-Takebe, Futami
Nagao, S.
Nagaoka, Kentaro
Nagaria, Pratik
Nagasawa, Hiromichi
Nagashima, Makoto
Nagatomo, Shigenori
Nagy, Istvan
Nahomi, Rooban Babyantony
Nair, Ashwin
Naito, Hisashi
Naito, Yuji
Najimi, Mustapha
Naka, Hiroaki
Naka, Kensuke
Nakabeppu, Yusaku
Nakagami, Futoshi
Nakagawa, Yoshimi
Nakaki, Toshio
Nakamura, Kazufumi
Nakamura, Kazuki
Nakamura, Kozo
Nakamura, Tsutomu
Nakanishi, Takeshi
Nakanishi, Toru
Nakashima, Kazuo
Nam, Kyoung Hee
Namiesnik, Jacek
Nan, Fan Hua
Nance, Christina L.
Nandrot, Emeline F.
Nanni, Loris
Nao, Dorit
Naoi, Makoto
Naoumkina, Marina
Narayana, Ashwatha
Nasopoulou, Constantina
Nateri, Abdolrahman Shams
Naumann, Todd A.
Navarra, Pierluigi
Navarro, Xavier
Nazzal, Sami
Nees, Matthias
Negi, Surendra S
Nemunaitis, John
Nesmelov, Yuri E.
Neumann-Haefelin, Christoph
Neuzil, Jiri
Nezhad, Amir Sanati
Ng, Jason
Ng, Kee Woei
Ng, Tzi Bun
Nguyen, Nam P.
Nguyen, Phuong
Nguyen, Thanh Vinh
Ni, Bing-Jie
Ni, Jiangfeng
Ni, Weiming
Nick, Peter
Nicolás, Francisco E.
Nicot, Christophe
Niessen, Hans W. M.
Niessen, Ludwig
Niessen, Markus
Nifosi, Riccardo
Nigris, De
Nijnik, Anastasia
Niku, Mikael
Nilsson, R. Henrik
Ning, Ke
Nino, Russo
Nishida, Shozo
Nishizaki, Tomoyuki
Nisman, Benjamin
Nisnevitch, Marina
Nissan, Aviram
Nixon, Joshua P.
Njoku, Dolores B
Nkenke, Emeka
Nobile, Mario
Noboru Katayama, Noboru
Nocentini, Giuseppe
Noguchi, Constance Tom
Nohe, Anja
Nojiri, Shunsuke
Nojiri, Takashi
Nolan, Christopher J
Nomiya, Takuma
Nomura, M.
Nonhebel, Heather M.
Noordzij, Walter
Nordin, Joel
Norman, Robert
Norman, Trevor R.
Norval, Mary
Novak, Johannes
Novak, Michael
Novo-Uzal, Esther
Nowak, Grazyna
Nowicka, Anna M.
Numakawa, Tadahiro
Nunes, Carla D.
Nunes, Leonel J. R.
Nunzio, Mattia Di
Nurmohamed, M T
Nyomba, B. L. Gregoire
Nyström, Andreas M.
Nyström, Kristina
O’Bryhim, Bliss E.
Oakeshott, John G.
Obana, A.
Oberthür, Markus
O'Brien, Katie M.
Ochiai, Tsuyoshi
Ochiya, Takahiro
Ocio, Enrique
Ock, Meesum
Ocorr, K.
Oesch, Franz
Ogata, Tadanori
Ogawa, Kumiko
Ogawa, Yoko
Ogino, Shuji
Ogura, Atsuo
Ogura, Shun-Ichiro
Oh, Sae-Ock
Oh, Sangsuk
Ohashi, Naro
Ohba, Shinsuke
Ohkohchi, Nobuhiro
Ohkura, N.
Ohmuraya, Masaki
Ohshima, Takashi
Ohta, Yoshiji
Ohta, Yuko
Oike, Yuichi
Ojeda, Manuel
Okaichi, Kumio
Okajima, Fumikazu
Okamoto, Masami
Okamura, Noboru
Okazaki, Toshiro
Oko, Richard
Okumoto, Sakiko
Okwumabua, Ogi
Oliveira, Pedro F.
Oliviera, Paulo
Olivieri, Fabiola
Olsson, Christian
O'Malley, Ronan
Omar, Syed Haris
O'meara, D. B.
Ondruschka, Benjamin
Ono, Kenji
Onori, Roberta
Ooi, Jenny
Oono, Youko
Oostenbrink, Chris
Oosterwijk, Egbert
Oottikkal, Shameema
Opstad, Trine B.
Orlandi, Augusto
Orozco, Jahir
Ortar, Giorgio
Orth, Kim
Ortlund, Eric A.
Osborn, Mark
Osbourn, Anne
O'Shea, Cormac
Oshima, Hayato
Oshitari, Toshiyuki
Osmond, Megan
Ospina Millán, Claudia Andrea
Osumi, Noriko
Otsuka, Hideaki
Ott, Ilka
O-Uchi, Jin
Ovissipour, Mahmoudreza
Owen, John
Oyama, Fumitaka
Ozaki, Toshinori
Ozeki, Yoshihiro
Ozen, Mustafa
Ozpolat, Bulent
Pa, Pai-Shan
Pace, Andrea
Pacifico, Severina
Paduch, Roman
Paek, Seung-Min
Pagani, Franco
Pagano, Joseph S.
Pajares, María A.
Palacios, José-Manuel
Palazzo, Alexander F.
Palma, Francesco
Palmer, Mike
Palmer, R.
Palmiro, Poltronieri
Palmisano, Giuseppe
Palomares, Eva Rufino
Palomo, G. Iván
Palomo-Ríos, Elena
Palstra, Thomas T. M.
Palumbo, Silvia
Pan, Hung-Chuan
Pan, Jingzhe
Pan, Tai-Long
Pan, Zhejun
Panchapakesan, Balaji
Panchenko, Maria V.
Pancsa, Rita
Pandanaboina, Sahitya Chetan
Pando, Concepción
Pañeda, Covadonga
Panek, Jaroslaw
Panfoli, Isabella
Pang, Alan Lap-Yin
Pang, Yi
Panov, Kostya
Pant, Deepak
Pantazis, Nicholas J.
Pantel, Jacques
Panteris, Emmanouil-Nikolaos
Pantin-Jackwood, Mary
Paolocci, Nazareno
Papaccio, GianPaolo
Papadopoulos, Vassilios
Papagianni, Maria
Papetti, Adele
Papi, Alessio
Paradiso, Angelo
Paraknowitsch, Jens Peter
Parales, Rebecca E.
Parameswaran, Narayanan
Pariente, J. A.
Parish, Roger W.
Parisi, Silvia
Park, Chail-Il
Park, Cheol Whee
Park, Cheon Seok
Park, Choon-Sik
Park, Chulhwan
Park, Eun-Jung
Park, Hoon Cheol
Park, Jang-Su
Park, Jeong-Sook
Park, Ji-Yeon
Park, Joong Kon
Park, Jung-Keug
Park, Junsoo
Park, Ky Young
Park, SungWoo
Park, Sang-Hyuk
Park, Se Hoon
Park, Se Pill
Park, Yoonkyung
Parnell, Jill A.
Pascual, Alberto
Pasinetti, Giulio M.
Pasqua, G.
Pastor, F. I. Javier
Patel, Ashok R.
Patel, Divya
Patel, Sandeep
Patel, Yashomati M.
Patrice, Thierry
Patruno, Marco
Patsenker, Eleonora
Pattison, David
Paulis, Maria
Paull, Robert E
Paull, Tanya T.
Paulose, Bibin
Pau-Roblot, Corinne
Pavanello, Sofia
Pavlov, Valeri
Payan-Carreira, Rita
Pazarentzos, Evangelos
Pedraza-Chaverri, José
Pedron, T.
Pei, Sung-Nan
Peijnenburg, Willie J. G. M.
Pék, Zoltán
Pelicci, Pier G.
Pellegrini-Giampietro, Domenico E.
Pelletier, Jerry
Pelosi, Paolo
Peluso, Marco E. M.
Pena, Romi N.
Peng, Ching-An
Peng, Guangyong
Peng, Shuping
Peng, Wen-Huang
Penn, Linda Z.
Pennypacker, Keith
Penzes, Peter
Peral, Belén
Pereira, G. R.
Pereira, Gislene
Pereira, Letricia Barbosa
Perez, Franck
Pérez-Figueroa, Andrés
Pérez-Gil, Jesús
Periyannan, Sambasivam
Perkins, Stephen J.
Perona, John J.
Perricone, Carlo
Perrine-Walker, F. M.
Perticone, Francesco
Péterfy, Miklós
Peterman, Erwin J. G.
Peters, Rene
Petersen, Bent
Petrey, Donald
Petro, Thomas M.
Petrochenko, Peter E.
Peula-García, José Manuel
Pfau, Jean C.
Phan, Manh-Huong
Philip, Nisha
Phillippi, Julie A.
Phillips, Joanna
Phillips, R. B.
Pi, Rongbiao
Picardo, Mauro
Piccardo, Arnoldo
Pichler, Martin
Pichon, Chantal
Picking, Wendy
Pickkers, Peter
Picton, Paul
Piedrahita, Jorge A.
Piekoszewski, Wojciech
Pierobon, Mariaelena
Pieters, Roland J.
Pietro, Roberta Di
Piga, Antonio
Pignol, David
Pihlanto, Anne
Piiper, Albrecht
Pilati, Camilla
Pilgaard, Linda
Pilkington, Suzanne M.
Pilotti, Silvana
Pineau, Pascal
Pineda, Teresa
Pinelli, Patrizia
Pinkett, Heather W.
Pinto, Alice
Pinton, Paolo
Pioszak, Augen
Pischetsrieder, Monika
Pissard, Serge
Pistrosch, Frank
Pittia, Paola
Piv, Roberta
Plackett, David
Planchet, Elisabeth
Plech, Tomasz
Plett, Jonathan
Plotkin, Lilian I.
Plough, Louis
Pluskal, Tomáš
Poblaciones, Maria J.
Poch, Michael
Podjarny, Alberto
Podlasek, Carol A.
Pognonec, Philippe
Pohanka, Miroslav
Poirier, Isabelle
Polefka, T. G.
Polesel, Jerry
Pollard, Kenneth M
Polyak, Stephen J
Polyak, Steven
Pommier, Suellen J.
Pondugula, Satyanarayana R.
Pongdor, Sandor
Ponikau, Jens U.
Pontiki, Eleni
Pook, Mark A.
Popanda, Odilia
Poppenberger, Brigitte
Poree, Lawrence R.
Porteu, F
Post, M. J.
Poulsen, Michael
Pouysségur, Jacques
Power, Greg
Pöyhönen, Laura
Poyner, David
Prat, Maria
Preti, Delia
Prézeau, Laurent
Priault, Muriel
Prieto-Hontoria, Pedro
Prigione, Alessandro
Pritchard, K. A.
Pritsch, Karin
Profirovic, J.
Prokai, Laszlo
Prokunina-Olsson, Ludmila
Prozialeck, Walter C.
Puga, Alvaro
Puigdomènech, Pere
Puiggalí, J.
Puiggalí, Jordi
Punta, Carlo
Purdue, Edward
Purisima, Enrico O.
Putz, Mihai
Qaderi, Mirwais M.
Qi, Xin
Qi, Yiping
Qiao, Ruirui
Qin, Zhiqiang
Qiu, Herric
Qu, Hui-Qi
Quadrilatero, Joe
Quaglino, Elena
Quan, Guoxing
Quesada, Víctor
Quigley, Eamon
Quigley, James
Quinlivan, Julie A.
Quiñones, Beatriz
Qureshi, Nilofer
Racz, I.
Radhakrishnan, Mala
Raffa, Vittoria
Ragoussis, Jiannis
Ragusa, Maria Antonietta
Rahaman, S. Ohidar
Rahman, Azizur
Rai, Brajesh K.
Rainer, Peter P
Rainey, Jan. K.
Raisman, Geoffrey
Rakotondrabe, Micky
Rakwal, Randeep
Ralph, Stephen J.
Ramachandran, Girish
Ramachandran, Srinivasan
Raman, Prabhu
Ramana, Kota V.
Ramani, Komal
Rameshwar, Pranela
Rämet, Mika
Ramji, Dipak P.
Ramón Y Cajal, Santiago
Ramos, Lourdes
Ramos, Paula S.
Ramos, Sonia
Ramotar, Dindial
Ramsden, Lawrence
Randall, Stephen K.
Randall, Troy D.
Randazzo, Paul A.
Ranganathan, Sarangarajan
Ranzato, Elia
Rao, Juluri R.
Rao, Mahendra Surendra
Rashotte, Aaron M.
Rasooly, Reuven
Rastegar, Mojgan
Rastogui, Gurdeep
Ratajczak, M. Z.
Rattan, Ramandeep
Ratti, Claudio
Raula, Janne
Rautava, Jaana
Rautio, Jarkko
Raviv, Z
Rawlings, Neil D.
Ray, S
Raychaudhuri, Subhadip
Rea, Stephen
Rebe, Cedric
Rebillard, Amélie
Recalcati, Maria Paola
Reddy, Umesh K.
Redfors, Björn
Redon, Christophe E.
Reese, R. N.
Reeve, Vivienne
Rehm, Markus
Reich, Reuven
Reid, Christopher W.
Reijns, Martin
Reinders, Joerg
Reitzel, Adam
Ren, Jian-Guo
Ren, Shuxin
Renella, Giancarlo
Renes, J.
Rengathavasi, Thavasi
Rengo, Giuseppe
Renner, Matthias
Renshaw, Stephen A.
Repo, Eveliina
Ress, Anna Lena
Reuter, Klaus
Reuter, Peggy
Reverberi, Massimo
Rexach, Jesús
Reyes-Moreno, Carlos
Reynisson, Jóhannes
Rezamand, Pedram
Rezvani, Khosrow
Rezzani, Rita
Rhee, Man Hee
Ribet, David
Ricciardelli, Carmela
Richardson, Stephen M
Rigano, Daniela
Rim, Kyung Taek
Rimbach, Gerald
Rinaldi, Carlos
Rinderer, Thomas E.
Rinehart, Joseph P.
Risling, Mårten
Ris-Stalpers, Carrie
Ritchie, Rebecca
Rittschof, Daniel
Rivas, Germán
Rivera, Jennifer
Rizos, Demetrios
Rizzuti, Bruno
Robben, Lars
Robert, Jacques
Roberts, Arthur G.
Roberts, Victoria A.
Robertson, R. Paul
Rocha, Eduardo Melani
Rochel, Natacha
Rochette, Jacques
Rockstroh, J. K.
Rödel, Franz
Rodeo, Scott A.
Rodger, James A.
Rodrigues, António S.
Rodríguez, Antonio Martínez
Rodríguez, Dra Soraya
Rodriguez-Lafrasse, Claire
Rodríguez-López, Julián
Roeder, Ryan K.
Roesner, Lennart M.
Rogers, Hilary
Rognan, Didier
Roh, Gu Seob
Roh, Sangho
Rohemail, Changhyun
Rohila, Jai
Rohira, Aarti
Rohrer, G. A.
Roitbak, Tamara
Rojas, Mauricio
Rojo, Enrique
Rolka, Krzysztof
Romalde, Jesús López
Romero, Alejandro
Rondanelli, Mariangela
Rong, Zhili
Rosado, Catarina
Rosato, Edoardo
Rosei, Enrico Agabiti
Rosell, Rafael
Rosellini, Daniele
Rosenberger, Peter
Rosenzweig, Derek
Ross, Ashley E.
Ross, M. K.
Rossello, Josep A.
Rossi, Derrick J.
Rossignol, Dan
Rossoll, Wilfried
Rotenstreich, Ygal
Roujeinikova, Anna
Rousseau, Philippe
Rovida, Ermanna
Rowe, Jasmine M.
Rowley, Ann
Roy, Shyamal K.
Roy, Stuart J.
Roychowdhury, Sanjoy
Rozek, Wojciech
Rubattu, Speranza
Rubin, Jeffrey
Rubin, Robert L.
Rucci, Nadia
Rudrabhatla, Sairam V.
Rudyk, Olena
Ruelland, Eric
Rühl, Martin
Ruiz, José
Ruiz-Feria, C. A.
Ruiz-Larrañaga, Otsanda
Ruiz-Narváez, Edward A.
Ruiz-Perez, Victor L.
Rupenthal, Ilva D.
Rupp, Matthias
Rus, Alma
Rusin, Aleksandra
Russell, Steven Thomas
Russo, Antonio
Russo, Giuseppina T.
Russo, Matteo A.
Rustad, Turid
Ryan, Julie L.
Ryan, Michael C.
Ryan, Michael J.
Rychert, Jenna
Rylott, Elizabeth L
Sa, Tongmin
Saba, Julie D.
Saba, Nabil
Sabbatini, Simona
Sacchetti, Lucia
Sadik, Riadh
Sadoghi, Patrick
Sadok, Walid
Saeed, Maythem
Sael, Lee
Sáez, Guillermo
Safi, Carl
Sah, Hongkee
Sahay, Bikash
Sahin, Özgür
Saielli, Giacomo
Saisho, Y.
Saito, Akira
Saito, Kuniaki
Sakai, Hideki
Sakamoto, Atsushi
Sakkas, Giorgos K.
Sako, Yasushi
Saku, Takashi
Sakurai, Yoshihiko
Sala, Gessica
Salamone, Adele
Salamonsen, Lois A.
Salerno, Aurelio
Salgado, A. J.
Salhia, Bodour
Salimnia, Hossein
Salinari, Serenella
Salinas, Julio
Salisbury, Travis
Saliu, Francesco
Salmaggi, Andrea
Salmon, Adam
Salsbury, Fred
Salter, Donald
Samavati, Lobelia
Samson, Leona D.
Samuil Umansky, Samuil
Samuolienė, Giedrė
Sánchez Alcázar, José
Sanchez, Diego H.
Sanchez, Jean-Yves
Sanchez, Manuel
Sánchez, María-José
Sandrin, Todd R.
Sangadala, Sreedhara
Sanmartín, Carmen
Sanna, Fabio
Sano, Natsumi
Sansotera, Maurizio
Santamaria, Rita
Santini, Catherine C.
Santos-Gallego, Carlos
Santulli, Gaetano
Sapi, Eva
Sarasini, Fabrizio
Sarropoulou, Virginia
Sarti, Paolo
Sasagawa, Toshiyuki
Sasayama, Takashi
Sato, Kenji
Sato, Masahiro
Sato, Satoshi B.
Sato, Takami
Sato, Tatsuo
Satoh, Masahiko
Satoh, Shigeru
Sattler, Wolfgang
Sauder, Christian
Sauerwein, Helga
Sauge, M.-H.
Saulnier-Blache, J. S.
Savard, Christian
Savic, J. M.
Saville, Barry
Sawers, R. Gary
Sayer, John A.
Sayers, Thomas J.
Sbardella, Gianluca
Scadding, Guy
Scarlett, Christopher
Scatena, Roberto
Schachter, Michael
Schaefer, Jeremy
Schall, Constance A.
Schapira, Matthieu
Scheibe, Renate J.
Scheiner, Steve
Scheinman, Robert
Schekman, Randy
Schellhaas, Elisabeth
Scherer, Günther
Schiffman, Jessica D.
Schild, Lorenz
Schiller, Jürgen
Schinke, Thorsten
Schlattner, Uwe
Schlee, Martin
Schleifenbaum, Frank
Schmalz, Hans-Guenther
Schmeiser, Heinz H.
Schmid, Kara
Schmidt, Manfred
Schmidt, Wolfgang
Schmidtke, Sarah J.
Schmitz, I.
Schneider, Anja
Schneider, Barbara
Schneider, Bernd
Schneider, Sabine
Schnellmann, Rick
Schoeftner, Stefan
Schönknecht, Gerald
Schreiber, Caroline
Schreyer, David
Schröder, Jörg
Schröder, Katrin
Schröder, Uwe
Schröder, Wolfgang P.
Schulz, Benjamin
Schulze, Joachim
Schuman, Thomas
Schüring, A. N.
Schuster, Bernhard
Schwarz, Michael
Schwean-Lardner, Karen
Schwerdt, Gerald
Scocchi, Marco
Scorziello, Antonella
Scott, Nigel H.
Scott, Stuart A.
Scotti, Carla
Scovassi, Anna Ivana
Sears, Dorothy D.
Sebastián, Rosa María
Seca, Ana
Seena, Sahadevan
Seenan, Chris
Segall, Jeffrey E.
Ségui, Bruno
Seidler, Norbert W.
Seifert, Roland
Seiffert, Sebastian
Seitz, Jan-Marten
Seki, Naohiko
Sekino, Masashi
Sekiya, Ichiro
Selan, Laura
Seligman, Paul A.
Selinski, Silvia
Sen, Shukdeb
SenBanerjee, Sucharita
Seo, Hak Soo
Seo, Shigemi
Seo, Young-Kwon
Sepúlveda, Maria S.
Serafini, G.
Sergeev, Igor N.
Serralheiro, Maria Luísa M.
Serrano, María
Serrano, Maria J.
Serrano-Ríos, Manuel
Sershen, H.
Seth, Arun
Sethi, Gautam
Setou, Mitsutoshi
Settanni, L.
Sevilla, Michael
Sevilla, Noemí
Sha, Zhe
Shabala, Sergey
Shafirovich, Vladimir
Shah, Javeed A.
Shaik, Shavali
Shaikhali, Jehad
Shan, Yibing
Shanmugam, Muruganandan
Shao, Huanjie
Shapiro, Arthur
Sharabi, Andrew
Shareck, F.
Sharma, Arun K.
Sharma, Avadhesh C.
Sharma, Jyotsna
Sharma, Kumar
Sharma, Mridula
Sharma, Om Prakash
Sharma, Rajendra
Sharman, Edward H.
Shavit, Jordan A.
Shavrukov, Yuri
Shaw, Gerry
Shaw, Jonathan G.
Shaw, Pang-Chui
She, Jin-Xiong
Shelp, Barry
Shen, Che-Kun James
Shen, Chwan-Li
Shen, Jie
Shen, Jinfeng
Shen, Kunwei
Shen, Wen
Sheng, Baiyang
Sheng, Yi
Sheng, Yuewei
Sherbet, G. V.
Sherer, Nathan
Sherman, Jonathan H.
Sherman, Louis
Shertzer, Howard G.
Shetty, Pavan K.
Shetty, Sreerama
Sheu, Ming-Thau
Shi, Hubing
Shi, Liang
Shi, Qian
Shi, Wenyuan
Shi, Yongquan
Shibasaki, Futoshi
Shibata, Ikuya
Shiea, Jentaie
Shieh, Dar-Bin
Shieh, Joseph
Shieh, K. R.
Shigemura, Yasutaka
Shih, J.-Y.
Shih, Ming-Chih
Shiina, Takashi
Shikano, Takahito
Shiku, Hiroshi
Shillcutt, Samuel
Shima, Jun
Shimada, Masayuki
Shimamura, Michio
Shimizu, Bun-Ichi
Shimizu, Shigeomi
Shimizu, Takahiko
Shin, Ki-Hyuk
Shintani, Yasushi
Shiota, Masaki
Shiraishi, Naru
Shiraishi, Takehiko
Shirasawa, K.
Shirasawa, Takugi
Shivapurkar, Narayan
Shmizu, Ken
Shoenfeld, Yehuda
Shoji, Tsubasa
Shonhai, A. Addmore
Shou, Weinian
Shrivastava, Indira
Shukla, Shruti
Shum, Anderson Ho Cheung
Shumway, Sandy
Shyu, Huey-Wen
Si, Ping
Siddall, Robert J. D.
Sidhaye, Venkataramana
Sieck, G. C.
Siegal, Gene P.
Sier, Cornelis F. M.
Sieuwerts, Anieta M.
Sifringer, Marco
Sigalotti, Luca
Signore, Michele
Silikas, Nick
Silljé, Herman H. W.
Silva, Bruna
Silva, Simone S.
Silva, Sn
Simal-Gándara, Jesús
Siman, Robert
Simeoni, Luca
Simeonov, Anton
Simmel, Friedrich
Simmons, Blake
Simò, Carolina
Simon, Adeline
Simonić, Jasmina
Simpson, Bradley S.
Simpson, David A. C.
Simpson, David C.
Sims, Ian M.
Sinclair, K. D.
Sinden, Richard
Singer, Cherie A.
Singh, Ashok K.
Singh, Ashutosh
Singh, Om V.
Singh, Guramrit
Singh, Kumud K.
Singh, Rakesh
Singh, S. K.
Singh, Sher
Singh, Vineet
Sinnecker, Daniel
Sinnett, Daniel
Sips, Patrick Y.
Siracusa, Laura
Siraki, Arno G.
Sirdah, Mahmoud
Siskind, Leah J.
Sissler, Marie
Sitharaman, Balaji
Siwek, Agata
Siwela, Muthulisi
Skaaby, Tea
Skeie, Bente Sandvei
Skipworth, Richard J. E.
Skøt, Leif
Slavov, Gancho
Sliz, Piotr
Slominski, Andrzej
Sluyter, R.
Smagghe, Guy
Smahi, Hadj-Rabia
Smart, Lawrence B.
Smith, Alan
Smith, L. Courtney
Smith, Stephen B.
Smith, Suzanne V.
Smith-Moritz, Andreia M.
Smits, Saskia L.
Smolock, Christopher J.
Snelling, Sarah J. B.
Sobczak-Thépot, Joëlle
Söderhäll, Kenneth
Soin, Navneet
Sokolov, Mykyta
Sola, Rosa
Solary, Eric
Solé, José García
Soliman, Mahmoud E. S.
Solinís, M. Ángeles
Soll, Dieter
Soma, Takehisa
Somers, Veerle
Song, Anren
Song, Guo-Qing
Song, Hae-Ryong
Song, Jinhua
Song, Jonathan
Song, Nam Woong
Sonkoly, Enikö
Sonkusare, Swapnil K.
Sonne, Susan C.
Soreq, Hermona
Sorg, Olivier
Sosa, Manuel
Souček, Pavel
Soulier, Jean
Sousa, Hugo
Spahn-Langguth, Hildegard
Spanier, Britta
Spear, Brett T.
Speicher, David W.
Speziale, Marisa
Spittau, Björn
Spivak, Graciela
Sprung, Carl N.
Sridhar, Radhakrishnan
Srivastava, Pramod K.
Srivatsan, Eri S.
Srivenugopal, Kalkunte
Stacchiotti, Alessandra
Staege, Martin S.
Staels, Bart
Stafford, Graham P.
Stamler, Jonathan S.
Stangl, Gabriele I.
Stanta, Giorgio
Stark, Benjamin C.
Starkey, Jonathan
Stassi, Giorgio
Staudacher, Alexander H
Stec, David E.
Stehle, Jörg H.
Steigedal, Magnus
Stein, Gary S.
Stein, Nils
Stein, Thor
Steinbach, William J.
Steinestel, Konrad
Steinmann, Eike
Stelzl, Ulrich
Stepensky, David
Stewart Jr, C. Neal
Stewart, Duncan J.
Stewart, Michael
Stick, Reimer
Stith, Brad J.
Stobiecki, Maciej
Stocco, Douglas M.
Stoecklin, Georg
Stone-Elander, Sharon
Stork, Björn
Storrie, Brian
Storto, Giovanni
Stote, K. S.
Stotz, Michael
Stracker, Travis H.
Strandvik, Birgitta
Strasser, Andrea
Streck, André F.
Streckbein, Philipp
Streit, Wolfgang R.
Strekowski, Lucjan
Stricker, Sigmar
Strle, Klemen
Strömme, Maria
Stronati, Laura
Strongin, Alex
Stulnig, Thomas M.
Stultz, Collin M.
Stumph, William E.
Stunes, Astrid Kamilla
Stura, Enrico A.
Sturm, Richard A.
Su, Chun-Li
Su, Jui-Hsin
Su, Jyan-Gwo J.
Su, Meitsz
Su, Yunchao
Sucher, Nikolaus J.
Suen, Garret
Suenaga, Hikaru
Suetsugu, Shiro
Sugasawa, Kaoru
Sugawara, Akira
Sugimoto, Masahiro
Sugimoto, Mitsushige
Sugiyama, Tetsuya
Sugumaran, Manickam
Suh, Jun Kyu
Suhm, Martin
Sui, Guangchao
Sui, Jing
Sukkar, Maria B.
Suman, Devi S.
Sumi, Shoichiro
Sun, Daochun
Sun, Fei
Sun, Genlou
Sun, Peilong
Sun, Raymond Wai-Yin
Sun, Shaogang
Sun, Zhihua
Sun, Zhonghua
Sundfeldt, Karin
Sunna, Anwar
Surai, P. F.
Surguchov, Andrei
Suri, Rominder P. S.
Sushruta, Koppula
Sussman, Daniel A.
Sutela, Suvi
Suzuki, A.
Suzuki, Akihiro
Suzuki, Hidekazu
Suzuki, Hitoshi
Suzuki, Kensaku
Suzuki, Masatoshi
Suzuki, Miho
Suzuki, Takuya
Suzuki, Yuichiro
Svanborg, Catharina
Svoboda, Miroslav
Swali, Angelina
Swann, Karl
Swarbrick, Crystall
Symons, J. David
Szablewski, Leszek
Szekeres, Thomas
Szeto, Yim Tong
Szlufik, Stanisław
Szumny, Antoni
Szyfter, Krzysztof
Tabata, Yasuhiko
Taberlay, Phillippa C.
Tada, Yayoi
Tadagavadi, Raghu
Taddei, Maurizio
Tagawa, Scott T
Taguchi, Tetsushi
Taira, Junsei
Takahashi, K. G.
Takahashi, Kazue
Takahashi, Naoyuki
Takahashi, Shigeru
Takahashi, Wataru
Takaki, Akinobu
Takase, Masayoshi
Takashiba, Shogo
Takayama, Koji
Takayama, Yoshiharu
Takebe, Naoko
Takeda, Makio
Takemori, Hiroshi
Takenaga, Keizo
Takeshita, Haruo
Takigawa, Nagio
Takumi, Shigeo
Talbot, Nicholas J.
Tall, Ben D.
Tamama, Kenichi
Tamura, Masaaki
Tamura, Tomohiro
Tan, Bao-Cai
Tan, Dun-Xian
Tan, Nguan Soon
Tanabe, Akiko
Tanaka, Hiroshi
Tanaka, Hiroyuki
Tanaka, Minoru
Tanaka, Mitsuru
Tanaka, Tim Hideaki
Tanase, Tomoaki
Tanavde, Vivek
Tang, Chih-Hsin
Tang, Haiyang
Tang, Liping
Tang, Wei
Tang, Xiaoli
Tang, Xiaoqing
Tang, Xuanming
Tang, Yuhong
Taniguchi, Akiyoshi
Taniguchi, Naoyuki
Taniguchi, Toshiyasu
Tanimoto, Kotaro
Tanji, Kunikazu
Tanner, J. A.
Tanner, N. Kyle
Tantillo, Dean
Tao, Wendong
Tappia, Paramjit S.
Tarantino, Giovanni
Tarca, Adi L
Tarr, D. Ellen
Tarro, Giulio
Tasciotti, Ennio
Tatlisumak, Turgut
Tattini, Massimiliano
Tatulian, Suren A.
Taube, Christian
Tavares, Isaura
Tawaraya, Keitaro
Tay, Samuel Sam Wah
Taylor, Allen
Taylor, John M
Taylor, Shirley M.
Taylor, Zachary S.
Taylor-Clark, Thomas
Tchikindas, Michael
Tcholakova, Slavka
Tedeschi, A.
Teeri, Teemu H.
Tegeder, Irmgard
Tegler, G.
Teicher, Beverly A.
Teijido, Oscar
Teismann, Peter
Teixeira, Cátia
Teixeira, M. C.
Teixeira, Natércia
Temperton, Ben
Tenbrock, Klaus
Tenenhaus, Mayer
Teng, Chieh-Lin
Terashima, Masaaki
Terauchi, Yasuo
Terentjev, Eugene M.
Tergaonkar, Vinay
Teriete, Peter
Terzaghi, William
Teschke, Rolf
Teta, Roberta
Tetley, Terry
Tettelbach, S. T.
Thalhammer, G.
Thamm, D. H.
Thangapazham, Rajesh L.
Tharreau, Didier
Thathiah, Amantha
Theel, Elitza
Theelen, Thomas
Theg, Steven M.
Thein, Tun-Linn
Theocharis, Stamatios
Theodoropoulos, George
Thérias, Sandrine
Thèvenod, Frank
Thi, Mia Mia
Thijssen, Dick H. J.
Thilmony, Roger
Thomas, David M.
Thomas, Olivier P.
Thomas, Sabu
Thomasova, Dana
Thomes, Paul
Thompson, Karl M.
Thompson-Snipes, Lu Ann
Thomsen, Hauke
Thomsen, Louiza Bohn
Thore, Stephane
Thorley, Andrew J
Thornburg, Kent
Thrasyvoulou, Andreas
Thyme, Summer B.
Tian, Linjie
Tian, Yinghua
Tichý, Aleš
Tiganescu, Ana
Tijssen, Peter
Tilden, Andrea R.
Timko, Michael Paul
Timofeyev, Valeriy
Ting, Kang
Tiribelli, Claudio
Tiwari, Sanjay
Tjong, S. C.
To, Kin-Ying
Tobajas, Montserrat
Toda, Kyoko
Tognini, Paola
Tohge, Takayuki
Tohyama, Chiharu
Tokino, Takashi
Tokita, Yoshihito
Toledo-Pereyra, Luis H.
Tomasetti, Marco
Tomatis, Maura
Tompa, Peter
Tonn, Jörg-Christian
Tonnabel, Jeanne
Tordjman, Sylvie
Torensma, Ruurd
Toriyama, Kinya
Törnquist, Kid
Török, Marianna
Toropov, Andrey A.
Torra, Ines Pineda
Torres-Aleman, Ignacio
Tosatto, Silvio
Tota, Bruno
Totsuka, Yukari
Touyz, Louis Z. G.
Toyama, Yusuke
Traas, Jan
Tran, Nhan L.
Treesukosol, Yada
Trendowski, Matthew
Tresguerres, Jesús
Trevisan, Andrea
Trif, Monica
Trincavelli, Maria Letizia
Trinchera, Marco
Trindle, Carl
Tripathi, Prateek
Troakes, Claire
Trosko, James E
Trotta, Francesco
Trucco, Massimo
Trujillo, Marco
Trummer, Arne
Tsai, Cheng-Fang
Tsai, Hui-Hsu Gavin
Tsai, Jyh-Ming
Tsai, Kuen-Jer
Tsai, Tsuimin
Tsakalof, A.
Tsantili, Anna
Tsao, Hsuan-Ming
Tsao, May N.
Tseng, Tai-Chung
Tsiplakou, E.
Tsirigotis, Panagiotis
Tsoleridis, Constantinos A.
Tsuboi, Koji
Tsuchimoto, Daisuke
Tsuda, Hiroyuki
Tsuji, Masahiro
Tsuji, Shoji
Tsujimoto, Takashi
Tsukamoto, Kazuhiro
Tsukiyama, Takuji
Tsuruya, Kazuhiko
Tudisco, Raffaella
Tung, Tao-Hsin
Turco, Gianluca
Turiel, Maurizio
Turner, David
Tuttolomondo, Antonino
Tyan, Yu-Chang
Tyystjärvi, Taina
Tzekov, Radouil
Uchinami, Hiroshi
Uchio, Yuji
Udomkun, Patchimaporn
Ueda, Hiroshi
Ueda, Koji
Uehara, Takeki
Ueki, Takeshi
Ueki, Tatsuya
Uematsu, Satoshi
Ueno, Hiroshi M.
Ueno, S.
Ueno, Takayuki
Ueoka-Nakanishi, H.
Ugalde, Unai
Uhal, Bruce D.
Ulivi, Paola
nalp, Aynur
Unosson, Erik
Upton, Jason W.
Urabe, Daisuke
Urbanc, Brigita
Urbanowicz, Ryan J.
Urlaub, Henning
Ursula, Eichenlaub-Ritter
Urszula, Kasprzykowska
Usatyuk, Peter V.
Usta, Berk
Usui, Soichiro
Usui, T.
Usui, Takeshi
Utzeri, Valerio Joe
Uwai, Koji
Vaidyanathan, Raman
Valacchi, Giuseppe
Valencia, Gregorio
Valentao, Patrícia
Valentová, Kateřina
Valero, M.
Valix, Marjorie
Valle, Beatriz
Valle, Luis J. Del
Valle, Mikel
Valsasina, Barbara
Valverde, Ángela M.
Van Agtmael, Tom
van Belkum, Marco
Van Berkel, Gary J.
van den Beucken, Jeroen J. J. P.
Van Bogaert, I. N. A.
Van Damme, Jo
van den Driesche, Sander
van der Kwast, Theodorus
van der Merwe, Margaretha
van Dijk, Jeroen P.
van Doorn, W. G.
Van Dyke, Knox
Van Dyke, Mark E.
Van Etten, Jaime
Van Gaal, Luc F.
van Geijlswijk, Ingeborg M.
van Hall, Thorblad
Van Hemelrijck, Mieke
Van Kessel, Ad Geurts
Van Leeuwen, Thomas
van Nieuw Amerongen, Geerten
Van Overbeek, Leonard S.
van Parys, Alexander
van Rheenen, Jacco
Van Seuningen, Isabelle
van Spriel, Annemiek B.
van Velthoven, Cindy T. J.
vande Velde, Christine
Vandebriel, Rob J
Vanden Eynde, Jean Jacques
Vandeweerd, J.-M.
Vankayala, Sai
Vankempen, Leon
Vanlerberghe, Greg C.
Vargas, W. A.
Vaskevich, Alexander
Vass, Imre
Vats, Purva
Vaudry, Hubert
Vautard, Frederic
Vavylonis, Dimitrios
Vaz, Deisi Altmajer
Vaz, Paula
Vaz, Roy J.
Vázquez, Julio
Vázquez-Prieto, Severo
Vedani, Angelo
Vega-Naredo, Ignacio
Veiga-Parga, Tamara
Vejnar, Charles
Velasco Ortega, Eugenio
Velkov, Tony
Vendrame, Martina
Venkannagari, Sreedhar
Vento, Renza
Venugopal, Jayarama Reddy
Verde, Ignacio
Verdoucq, Lionel
Vermeulen, Louis
Veronesi, Bellina
Vetter, Stefan W.
Vetvicka, Vaclav
Vicente, Laura
Vickers, Mark
Vidal, Marcos
Videira, Mafalda
Videira, Paula A.
Vieira, Alexandre
Viggiano, Davide
Vigodner, Margarita
Vihinen, Mauno
Villa, Erica
Villaboa, Nuria
Villalobo, Antonio
Villoutreix, Bruno O.
Vincent, Bourret
Vinggaard, Anne Marie
Vingtdeux, Valérie
Vinnedge, Lisa Privette
Virgilio, Francesco
Virtanen, Kirsi A.
Virtanen, Sannakaisa
Viscido, Angelo
Viscomi, Maria Teresa
Vitale, Giovanni
Vitetta, Luis
Vitorino, Rui
Vizcaychipi, Marcela P.
Vlachonasios, K. E.
Vladimirov, Vladimir I.
Vlahou, Antonia
Voelkers, Mirko
Vogel, Ulla
Voll, Lars
Von Knethen, Andreas
von Mikecz, Anna
Von Strandmann, Elke Pogge
Vonnahme, K. A.
Vora, Gary
Vornam, Barbara
Vougioukalakis, Georgios C.
Vreede, Jocelyne
Vreeken, Rob J.
Vriend, J.
Vrtala, Susanne
Wada, Keiko
Wadehra, Madhuri
Wagner, David
Wahl, M. A.
Wainwright, Mark
Wallace, Daniel
Wallace, Lisa
Wallentin, Lars
Walley, Andrew J.
Wallis, John D.
Walters, Kirsty A.
Wan, Lei
Wanasundara, Janitha
Wang, Bo-Cheng
Wang, Chenguang
Wang, Chengzhong
Wang, Chi Chiu
Wang, Chi-Young
Wang, Chrong-Reen
Wang, Dan
Wang, Dong
Wang, Guanyu
Wang, Hao
Wang, Haochao
Wang, Hsei-Wei
Wang, Hua
Wang, Jian
Wang, Jianbin
Wang, Ju-Ming
Wang, Jun
Wang, Junjie
Wang, Kai
Wang, L.
Wang, Leo D.
Wang, Li-Yu
Wang, Mingyi
Wang, Peng George
Wang, Qian
Wang, Qi-En
Wang, Richard R.-C.
Wang, Rui
Wang, S.-W.
Wang, Shanfeng
Wang, Shaobin
Wang, Shao-Chun
Wang, Shaomeng
Wang, Tzu-Hao
Wang, Tzu-Wei
Wang, Wen-Ching
Wang, Xiang-Dong
Wang, Xiaobin
Wang, Xiaoshi
Wang, Yibin
Wang, Yin
Wang, Ying
Wang, Yongyu
Wang, Yue
Wang, Yue-Hu
Wang, Yuemin
Wanke, Dierk
Ward, Peter A.
Wardrop, Duncan J.
Waris, Gulam
Washio, Kenji
Wass, Mark N.
Watanabe, Akihiko
Watanabe, Kenichi
Watanabe, Naoki
Watanabe, Toru
Watkins, Harriet
Watson, Keith G.
Watters, J. J.
Watters, Michael K.
Watts, Jennifer L.
Webb, Paul
Weber, Christopher
Weber, Jost
Ween, Miranda
Weerapreeyakul, Natthida
Weetman, David
Wei, Alexander
Wei, Gang
Wei, Jing
Wei, Kuo-Chen
Wei, Po-Li
Wei, Ting
Weickert, Martin O.
Weigel, R. J.
Weisberg, Ellen
Weisleder, Noah
Wellik, Deneen M.
Weltzien, Finn-Arne
Wen, C. M.
Wendel, Hans Peter
Weng, Ching-Feng
Weng, Honglei
Weng, Leiyun
Weng, Mao-Lun
Wennemuth, Gunther
Wennerber, Ann
Wensman, Jonas Johansson
Weretilnyk, Elizabeth
Wesolowski, Robert
Westenfelder, Christof
Westgate, Gill
Weston, Leslie
Weyand, Simone
Whan, Ahn J. I.
Wheeler, Korin E.
White, Anthony
White, Kenneth N.
White, P. Lewis
Whitelegge, Julian P.
Whiteson, Katrine
Whittall, Justen B.
Whyard, Steve
Wicherek, Lukasz
Wickramasinghe, Vihandha O.
Wielbo, Jerzy
Wiemhöfer, H.-D.
Wigle, J. T.
Wilding, Craig
Wiley, James S.
Wilgus, T. A.
Wilkinson, Gavin
Willem, Michael
Williams, M.
Williams, Tiffany
Wilmarth, Phillip
Wilson, Bill
Wilson, D. B.
Wilson, Joyce A.
Wilson, Lee D.
Wilson, Vincent L.
Winkler, David A.
Winkler, J.
Wirgin, Isaac
Wirsig-Wiechmann, Celeste R.
Wislet-Gendebien, S.
Wisniewski, Jacek
Witt-Enderby, Paula A.
Witting, Paul
Wittmann, Jürgen
Wojcik, Andrzej
Wojdyło, Aaneta
Wolf, Jennifer Moriatis
Wolf, Matthias
Wolf-Watz, Magnus
Woloszyk, Anna
Wolthers, Mariette
Won, Chong Hyun
Won, Seok Joon
Wong, Anderson On Lam
Wong, Boon-Seng
Wong, Carmen Chak-Lui
Wong, Chi-Ming
Wong, Dominic W. S.
Wong, Ferranti
Wong, Garry
Wong, Ka-Leung
Wong, Kenneth K. Y.
Wong, Liang-Fong
Wong, Melanie
Woo, Eui-Jeon
Woo, Sun-Hee
Woods, Alisa G.
Woods, Christopher J.
Wortham, Noel C.
Woźniakowski, G
Wright, Casey M.
Wright, Catherine
Wu, Alan Hb
Wu, Bing
Wu, Chih-Hsiung
Wu, Jaw-Ching
Wu, Jen-Leih
Wu, Jian-Ping
Wu, Ji'en
Wu, Jiunn-Ren
Wu, Lawren C.
Wu, Lianfeng
Wu, Li-Chen
Wu, M.
Wu, Meng
Wu, Ming-Jiuan
Wu, Mousheng
Wu, Qiangen
Wu, Shih-Hsiung
Wu, Shiyong
Wu, Shuang
Wu, Shu-Yii
Wu, William Ka Kei
Wu, Xiaofeng
Wu, Yi-Jun
Wu, Yu-Jen
Wuertz, Karin
Wunder, Stephanie
Wyss, Christine
Xi, Lei
Xi, Yaguang
Xia, Tian
Xia, Ying
Xiao, Li
Xiao, Xiangshu
Xie, Lei
Xiong, Ye
Xiong, Yuquan
Xu, Baoshan
Xu, Jinbo
Xu, Keli
Xu, Lin
Xu, Ning
Xu, Xiaoming
Xu, Xingshun
Xu, Yi-Jun
Yaegaki, Ken
Yaguch, Tomonori
Yahara, Shoji
Yakovlev, Igor A.
Yakushi, Toshiharu
Yamada, Hiroyuki
Yamada, Tesshi
Yamaguchi, A.
Yamaki, Koji
Yamamoto, Kohji
Yamamoto, Norio
Yamamura, Soichiro
Yamanushi, Tomoko T.
Yamashita, Naomi
Yamauchi, Takahiro
Yamawaki, Hideyuki
Yammani, Raghunatha R
Yan, Elsa C. Y.
Yan, Liang-Jun
Yan, Xuehai
Yanase, K.
Yang, Bin
Yang, Chia-Ning
Yang, Ding-Yah
Yang, Fan
Yang, Hyun Ok
Yang, Jian
Yang, Kwangmo
Yang, Li
Yang, Lirong
Yang, Liu
Yang, Muh-Hwa
Yang, Qiwei
Yang, Rong Sen
Yang, Rongze
Yang, Wanling
Yang, Wannian
Yang, Yong
Yang, Yongguang
Yang, Yuedong
Yang, Yu-Min
Yang, Zhi-Min
Yang, Zujun
Yaniv, Moshe
Yano, Shozo
Yarema, Kevin
Yasuhara, Takao
Yasui, Akira
Yates, Edwin A.
Yates, Steven
Yazawa, Takashi
Yazlovitskaya, Eugenia M.
Ye, Richard D.
Yeh, Jui-Ming
Yeh, Jwu-Lai
Yeh, Shu-Lan
Yen, Feng-Lin
Yen, Men-Luh
Yennu-Nanda, Vashisht G.
Yeung, Kelvin
Yeung, Sai-Ching Jim
Zhao, Yilei
Yin, Lei
Yin, Yulong
Yokoi, Tsuyoshi
Yokoyama, Yoshihito
Yomo, Tetsuya
Yonekura, Shinichi
Yoo, Seong Ho
Yoo, Yeong-Min
Yoon, Hwan Su
Yoon, Sang Pil
York, William S.
Yoshida, Katsunori
Yoshida, Manabu
Yoshida, Takashi
Yoshida, Yasuhiro
Yoshidome, H.
Yoshiki, Katayama
Yoshimura, Akihiko
Yoshimura, Kazuya
Yoshimura, Y.
Yoshinari, Tomoya
Yoshioka, Yasuo
Yoshitomi, Hiroyuki
Yoshizaki, Yumiko
You, Liang
You, Mingxu
You, Zhaoyang
Youn, Buhyun
Youn, Yu Seok
Young Hyun, Choi
Young, David A
Young, Howard
Young, K. C.
Young, Matthew R.
Young, Terry-Lynn
Yousefi, Behrooz H.
Yousefi, Shida
Yu, Aimin
Yu, Cheng-Chia
Yu, Eileen Hao
Yu, Il Je
Yu, Lijian
Yu, Mengxiao
Yu, Shihui
Yu, Xiong
Yu, Yan Ping
Yu, Yinghua
Yu, Yong-Ming
Yuan, Shinsheng
Yuba, Eiji
Yue, Genhua
Yue, Patrick Ying Kit
Yuh, Chiou-Hwa
Yun, Jong Won
Yung, Wing-Ho
Zabel, Brian A.
Zachar, V.
Zacharias, Martin
Zafari, A. Maziar
Zagrovic, Bojan
Zahradka, Peter
Zakhartchouk, Alexander
Zakrzewic, Anna
Zalapa, Juan Ernesto
Zamaratskaia, Galia
Zanardi, Franca
Zanetti, Michela
Zannini, Emanuele
Zargaran, Massoumeh
Zarnowski, Tomasz
Zarogoulidis, Paul
Zarra, Ignacio
Zarrelli, Armando
rský, Viktor
Zavan, Barbara
Zehendner, Christoph M.
Zeisel, M.
Zeitzer, Jamie M.
Zelikoff, Judith
Zenarruzabeitia, Olatz
Zeng, Qingning
Zenoni, Sara
Zeschnigk, Michael
Zewail, H.
Zhai, Guangju
Zhai, Haiyan
Zhang, Chengfei
Zhang, Chris
Zhang, Dingguo
Zhang, Fazhi
Zhang, Gao
Zhang, Ge
Zhang, Haoqian
Zhang, Huiming
Zhang, Jiangying
Zhang, Lei
Zhang, Li
Zhang, Miao
Zhang, Mingjun
Zhang, Mingming
Zhang, Peng
Zhang, Qiang
Zhang, Ruiwen
Zhang, Shao-Wu
Zhang, Shihong
Zhang, Shuai
Zhang, Weizhen
Zhang, Weizhou
Zhang, Wenheng
Zhang, Wentao
Zhang, Wenzheng
Zhang, Xiao-Bing
Zhang, Xu Dong
Zhang, Xuchen
Zhang, Xuguang
Zhang, Yanmin
Zhang, Yao
Zhang, Yaqing
Zhang, Yi
Zhang, Yuan-Ye
Zhang, Yuanyuan
Zhang, Zhaolei
Zhang, Zhihong
Zhao, Boxin
Zhao, Chenguang
Zhao, Chunfeng
Zhao, Lan
Zhao, Lixia
Zhao, Q.
Zhao, Shou-Jing
Zhao, Ting C
Zhao, Ying-Yong
Zhen, Gehua
Zheng, Liangyu
Zheng, Shilong
Zheng, Wenguang
Zheng, Xiaoqi
Zhou, Carol L. Ecale
Zhou, Fei
Zhou, Guo-Ping
Zhou, Hongyu
Zhou, Huan
Zhou, Huitong
Zhou, Kequan
Zhou, Man
Zhou, Ping
Zhou, Qingxiang
Zhou, Shuanhu
Zhou, Xuan-Wei
Zhou, Yong-Hong
Zhou, Zhuan
Zhu, Donghui
Zhu, Qiang
Zhu, Yanming
Zhu, Yimin
Zhuo, Jia L.
Zienolddiny, S.
Zignego, Anna Linda
Zimmerman, Matthew C.
Zimmermann, Katharina
Zingarelli, Basilia
Zink, Daniele
Zoccali, Carmine
Zoetendal, Erwin
Zöller, Margot
Zorzano, Antonio
Zou, Hejian
Zou, Shengli
Zou, Xueqing
Zuccato, Chiara
Zucman-Rossi, Jessica
Zuhl, Micah
Zumsteg, Anna
Zuo, Li
Zuo, Yi
Zurek, Eva

